# Spatiotemporal whole-brain activity and functional connectivity of melodies recognition

**DOI:** 10.1093/cercor/bhae320

**Published:** 2024-08-07

**Authors:** Leonardo Bonetti, Elvira Brattico, Francesco Carlomagno, Joana Cabral, Angus Stevner, Gustavo Deco, Peter C Whybrow, Marcus Pearce, Dimitrios Pantazis, Peter Vuust, Morten L Kringelbach

**Affiliations:** Center for Music in the Brain, Department of Clinical Medicine, Aarhus University & The Royal Academy of Music, 8000 Aarhus/Aalborg, Denmark; Centre for Eudaimonia and Human Flourishing, Linacre College, University of Oxford, OX39BX Oxford, United Kingdom; Department of Psychiatry, University of Oxford, OX37JX Oxford, United Kingdom; Center for Music in the Brain, Department of Clinical Medicine, Aarhus University & The Royal Academy of Music, 8000 Aarhus/Aalborg, Denmark; Department of Education, Psychology, Communication, University of Bari Aldo Moro, 70121 Bari, Italy; Center for Music in the Brain, Department of Clinical Medicine, Aarhus University & The Royal Academy of Music, 8000 Aarhus/Aalborg, Denmark; Center for Music in the Brain, Department of Clinical Medicine, Aarhus University & The Royal Academy of Music, 8000 Aarhus/Aalborg, Denmark; Centre for Eudaimonia and Human Flourishing, Linacre College, University of Oxford, OX39BX Oxford, United Kingdom; Life and Health Sciences Research Institute (ICVS), School of Medicine, University of Minho, 4710-057 Braga, Portugal; Center for Music in the Brain, Department of Clinical Medicine, Aarhus University & The Royal Academy of Music, 8000 Aarhus/Aalborg, Denmark; Centre for Eudaimonia and Human Flourishing, Linacre College, University of Oxford, OX39BX Oxford, United Kingdom; Computational and Theoretical Neuroscience Group, Center for Brain and Cognition, Universitat Pompeu Fabra, 08018 Barcelona, Spain; Institució Catalana de la Recerca i Estudis Avançats (ICREA), Passeig Lluís Companys 23, Barcelona, Spain; Semel Institute for Neuroscience and Human Behavior, University of California, Los Angeles, 90095 Los Angeles, CA, United States; Center for Music in the Brain, Department of Clinical Medicine, Aarhus University & The Royal Academy of Music, 8000 Aarhus/Aalborg, Denmark; McGovern Institute for Brain Research, Massachusetts Institute of Technology (MIT), 02139 Cambridge, MA, United States; Center for Music in the Brain, Department of Clinical Medicine, Aarhus University & The Royal Academy of Music, 8000 Aarhus/Aalborg, Denmark; Center for Music in the Brain, Department of Clinical Medicine, Aarhus University & The Royal Academy of Music, 8000 Aarhus/Aalborg, Denmark; Centre for Eudaimonia and Human Flourishing, Linacre College, University of Oxford, OX39BX Oxford, United Kingdom; Department of Psychiatry, University of Oxford, OX37JX Oxford, United Kingdom

**Keywords:** memory, sequence recognition, brain spatiotemporal dynamics, functional connectivity, magnetoencephalography (MEG)

## Abstract

Music is a non-verbal human language, built on logical, hierarchical structures, that offers excellent opportunities to explore how the brain processes complex spatiotemporal auditory sequences. Using the high temporal resolution of magnetoencephalography, we investigated the unfolding brain dynamics of 70 participants during the recognition of previously memorized musical sequences compared to novel sequences matched in terms of entropy and information content. Measures of both whole-brain activity and functional connectivity revealed a widespread brain network underlying the recognition of the memorized auditory sequences, which comprised primary auditory cortex, superior temporal gyrus, insula, frontal operculum, cingulate gyrus, orbitofrontal cortex, basal ganglia, thalamus, and hippocampus. Furthermore, while the auditory cortex responded mainly to the first tones of the sequences, the activity of higher-order brain areas such as the cingulate gyrus, frontal operculum, hippocampus, and orbitofrontal cortex largely increased over time during the recognition of the memorized versus novel musical sequences. In conclusion, using a wide range of analytical techniques spanning from decoding to functional connectivity and building on previous works, our study provided new insights into the spatiotemporal whole-brain mechanisms for conscious recognition of auditory sequences.

## Introduction

Research in neuroscience of music has rapidly grown in the past decades (Münte et al. 2002; Koelsch et al. 2004; Koelsch et al. 2019; Pando-Naude et al. 2021). Indeed, since music is an art that acquires meaning through the combination of its constituent elements extended over time (Cooke 1959; Peretz and Zatorre 2003), it provides as an excellent tool for investigating the brain’s temporal dynamics (Münte et al. 2002; Koelsch et al. 2004; Koelsch et al. 2019; Pando-Naude et al. 2021).

Several studies have focused on the processing of sounds and revealed the primary role of the auditory cortex (Näätänen et al. 1978; Warrier et al. 2009; Brattico and Pearce 2013). These investigations uncovered the early, well-known components of the event-related potential/field (ERP/F) that occurs in response to sounds, such as the N100, mismatch negativity, and P3a (Näätänen et al. 1978; Näätänen et al. 2007). Additional studies broadened these investigations by employing more complex musical stimuli and analytical techniques. For instance, it has been widely shown that music processing evokes activity in brain networks connected to emotions (Koelsch 2014). A classic study by Blood and Zatorre (2001) revealed that listening to pleasurable music was associated with a burst of activity in brain areas related to pleasure and reward such as amygdala, orbitofrontal cortex, ventral medial prefrontal cortex, and striatum. In addition to neural regions connected to emotions, it has been shown that music processing recruits motor areas of the brain such as the supplementary motor cortex, basal ganglia, and cerebellum, which are responsible for tracking rhythm and musical beat, as described by Kotz et al. (2018) and Nozaradan et al. (2017).

Music listening has been investigated not only in terms of brain activity but also considering the associated functional connectivity between brain areas and its relationship with musical expertise. For instance, Alluri et al. (2015) investigated musicians and non-musicians while they were listening to music. They found that musicians had stronger connectivity than non-musicians between supplementary motor area (SMA) and ventromedial and ventrolateral cerebral and cerebellar affective regions. Differently, non-musicians compared to musicians showed stronger connectivity between subcortical regions only. In an electroencephalography (EEG) study, Bhattacharya and Petsche (2005) reported enhanced gamma band–phase synchrony when participants with musical expertise listened to music.

Notably, music has also been employed to investigate brain mechanisms connected to memory. Beyond the strong emotional content evoked, music contains complex logical, hierarchical structures (Cooke 1959), yielding to meaningful messages and information that can be encoded and recognized. Along this line, several studies employed functional magnetic resonance imaging (fMRI) and paradigms involving music memorization and evaluation of specific melodic features.

For instance, Gaab et al. (2003) measured the brain activity while participants were asked to compare different simple melodic sequences. When participants successfully carried out the task, their brain activity was mainly observed in the superior temporal, superior parietal, posterior dorsolateral frontal, and dorsolateral cerebellar regions and supramarginal and left inferior frontal gyri. In another classic study (Zatorre et al. 1994), authors dissociated the perceptual analysis of melodies from the pitch comparison of particular tones. They revealed that the former process was associated with activity in the right superior temporal cortex, while the latter mainly involved the right prefrontal cortex. A more recent study by Kumar et al. (2015) highlighted the crucial role of the primary auditory cortex, inferior frontal gyrus and hippocampus underlying an auditory working memory (WM) task consisting of maintaining a series of single sounds. Remarkably, the authors showed that not only the activity but also the connectivity between these three areas were linked to the successful completion of the task.

Auditory memory has also been studied by employing magnetoencephalography (MEG), which is beneficial to detect the fast-scale brain activity associated with memory tasks. A large corpus of studies investigated fast preattentive neural responses, implying the existence of sensory auditory memory, namely, N100 and MMN (Bonetti et al. 2021a; Bonetti, Carlomagno, et al. 2022b; Bonetti et al. 2018; Bonetti et al. 2017; Näätänen et al. 2007). Additional research has investigated auditory memory by using more complex tasks and experimental designs. In another study, Albouy et al. (2017) investigated the brain activity underlying memory retention. The authors showed that theta oscillations in the dorsal stream of the participants’ brain predicted their abilities to perform an auditory WM task that consisted of maintaining and manipulating sound information.

Recently, we expanded on this research by investigating the brain mechanisms underlying long-term encoding and recognition of musical sequences. First, we studied the activity and connectivity in the healthy brain underlying the encoding of single sounds forming a highly structured musical prelude (Bonetti et al. 2021b). Our results showed that the first 220 ms of sound processing were associated with a wide network of functionally connected brain areas. Notably, while the brain activity was mainly observed for the primary and secondary auditory cortex and insula, functional connectivity analysis returned a larger picture of equally central brain areas, including not only the auditory cortex and insula but also the hippocampus, basal ganglia, cingulate gyrus, and frontal operculum. These results showed the importance of conducting fast-scale analysis on both activity and functional connectivity when studying encoding of sounds.

Second, we conducted two studies specifically focused on investigating the brain mechanisms underlying recognition of previously memorized melodies taken from the whole musical piece used in our previous study on sound encoding (Bonetti et al. 2021b). In the first of these two studies, we focused on the brain activity filtered in two different frequency bands to reveal that the single sounds forming the melody were connected to local and rapid (2 to 8 Hz) brain processing, while the whole sequence was linked with concurrent global and slower (0.1 to 1 Hz) processing involving a widespread network of brain regions (Bonetti et al. 2022a). Importantly, this study compared previously memorized musical sequences versus completely novel sequences and only focused on univariate analysis based on the ERF generated by the stimuli in two specific frequency bands. Thus, no functional connectivity nor broadband multivariate pattern analysis was conducted. In the second study, we utilized the same experimental paradigm but implemented key modifications to the stimuli. Specifically, we altered the musical pace, with each sound lasting 350 ms compared to the 250 ms described in Bonetti et al. (2022a). Additionally, novel melodies were created by keeping the first sound identical to the previously memorized ones and systematically altering the subsequent sounds (e.g. changing all the sounds from the second tone onward, or from the third, fourth, or only the fifth tone). This approach allowed us to study recognition in a different musical tempo and investigate the brain mechanisms underlying the prediction error generated by the novel stimuli, which presented systematic changes from the original, memorized melodies. In this study, we also employed a comprehensive array of analyses, including broadband multivariate pattern analysis.

Building on our previous research, the current study uses the same data reported in Bonetti et al. (2022a) and aims to both replicate some of our previous results and expand them by investigating novel specific, yet relevant details.

First, we aim to test whether the recognition of previously memorized versus novel melodies is associated to changes only in the whole-brain activity or also in the functional connectivity patterns measured during the task.

Second, we compare the brain networks revealed by the functional connectivity analysis with those reported for sound encoding in Bonetti et al. (2021b). This comparison is particularly meaningful since, in the current study, we used a different dataset obtained from the same participants as in Bonetti et al. (2021b).

Third, we use temporal generalization in multivariate pattern analysis to study how brain patterns can be generalized over time. In Bonetti et al. (2024), we demonstrated that recognizing previously memorized versus systematically varied musical sequences produced stable brain patterns recurring over time for the entire duration of the musical sequence. In fact, in that study, the brain responses to each sound in the memorized and novel sequences were consistently similar, indicating that the brain monitored each sound, confirmed predictions when they matched the memory trace, and detected errors when they did not. In this study, we instead compare previously memorized melodies to completely novel ones (i.e. not varied after some sounds) to assess if the brain patterns of differential activity remain stable over the entire musical sequence as in Bonetti et al. (2024) or diverge.

Finally, based on previous evidence on the relationship between cognitive abilities, musical expertise, and music perception (Herholz et al. 2008; Bonetti and Costa 2016, 2019; Criscuolo et al. 2019; Criscuolo et al. 2022; Fernández-Rubio et al. 2023), in this study, we also assess whether WM and musical expertise modulate the brain activity underlying recognition of previously memorized music.

## Materials and methods

### Overview of the experimental design and of the data analysis pipeline

**Fig. 1:**
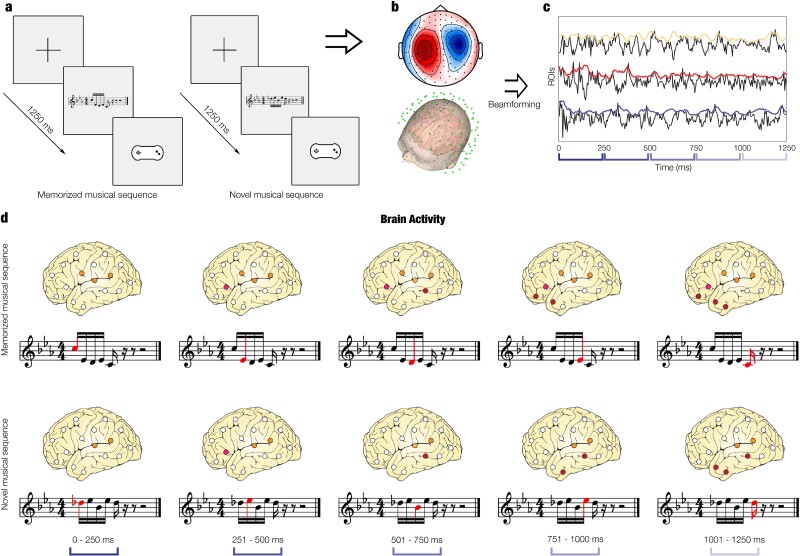
Experimental design and analysis methods. a) Graphical schema of the old/new paradigm. One at a time, several five-tone musical sequences (melodies) were presented. These could belong either to the prelude that participants had previously listened to (memorized musical sequence, “old”) or could be novel musical sequences (“new”). In this figure, we depicted at first an example of a memorized musical sequence (“old”) sequence (left, 2nd square) with the relative response pad that participants used to state whether they recognized the excerpt as “old” or “new” (left, 3rd square). Then, we depicted an example of novel musical sequence (“new,” right, 2nd square). The total number of trials was 80 (40 memorized and 40 novel musical sequences), and their order was randomized. b) We collected, preprocessed, and analyzed MEG sensor data by employing multivariate pattern analysis and MCS on univariate tests. c) We beamformed MEG sensor data into source space, providing time series of activity originating from brain locations. d) We studied the source brain activity underlying the processing of each tone of the musical sequences for both experimental conditions.

In this study, we wanted to characterize the fine-grained spatiotemporal dynamics of whole-brain activity and functional connectivity during recognition of previously memorized auditory sequences. In brief, during a session of MEG, 70 participants listened to the full prelude in C minor BWV 847 composed by Bach and tried to memorize it as much as possible. As depicted in Fig. 1a and Figure SF1, participants were then presented with short musical melodies corresponding to excerpts of Bach’s prelude and carefully matched novel musical sequences and were asked to indicate whether each musical excerpt was extracted from Bach’s prelude or was a novel melodic sequence.

The analysis pipeline used in this study is partly illustrated in Fig. 1b and described in detail in the following paragraphs, according to recommendations offered by Gross et al. (2013) and Pernet et al. (2020). This focused on extracting results using three main measures of brain functioning: (i) MEG sensor space activity, (ii) beamformed source localized activity, (iii) static source localized connectivity.

We computed a vast array of analyses for two reasons: to strengthen the reliability of our results by obtaining converging findings from different analytical approaches (e.g. multivariate pattern analysis and Monte Carlo simulation [MCS] on univariate tests) (i); to integrate our brain activity analysis with functional connectivity investigations. The aim of the second method was to detect the relationship and communication between brain areas and not their mere activity in response to our musical stimuli (ii).

First, we used multivariate pattern analysis and MCSs on univariate tests of MEG sensor data. Second, we were interested in finding the brain sources of the observed differences and therefore we reconstructed the sources of the signal using a beamforming algorithm (Fig. 1c) to track the brain activity related to each tone of the musical sequences (Fig. 1d). Third, complementing our brain activity results, we computed evoked-responses functional connectivity between brain regions. We calculated the static functional connectivity by computing Pearson’s correlations between the envelopes of each pair of brain areas, focusing especially on whole-brain connectivity and degree centrality of brain regions.

### Participants

By computing a vast array of novel analyses, this study expands on our previous works on the brain mechanisms underlying music encoding and recognition (Bonetti et al. 2022a; Bonetti et al. 2021b; Bonetti et al. 2024; Bonetti et al. 2024; Fernandez-Rubio et al. 2022; Fernández-Rubio et al. 2022). To ensure full transparency, we provide the following detailed information. With regard to the current study, Bonetti et al. (2024) refer to a different dataset with a distinct set of novel melodies (and musical tempo) obtained from a completely different sample. Bonetti et al. (2021a) utilized a different dataset based on sound encoding in the same participants as the current study. Fernandez-Rubio et al. (2022) and Fernández-Rubio et al. (2022) used different datasets based on music recognition in nearly the same participants as the current study. Finally, Bonetti et al. (2022a) employed the same dataset as the current study but focused on different analyses: univariate tests on brain activity in specific frequency bands. The current study employs instead broadband multivariate pattern analysis, broadband univariate tests, and functional connectivity in selected frequencies.

The sample of this study consisted of 70 volunteers who performed an “old/new” auditory paradigm. All participants came from different Western countries and lived in Denmark at the time of the experiment. Thirty-six of them were males and 34 were females (age range: 18 to 42 years old, mean age: 25.06 ± 4.11 years). Since our experiment involved a musical piece usually played by classical pianists, we recruited 23 classical pianists (13 males and 10 females, age range: 18 to 34 years old, mean age: 24.83 ± 4.10 years old), 24 non-pianist musicians (12 males and 12 females, age range: 19 to 42 years old, mean age: 24.54 ± 4.75), and 23 non-musicians (11 males and 12 females, age range: 21 to 35 years old; mean age: 25.86 ± 3.34). The sample regarding functional connectivity analysis slightly differed (three participants had to be discarded due to technical problems during acquisition) and consisted of 67 participants (34 males and 33 females, age range: 18 to 42 years old, mean age: 25.00 ± 4.18 years). Specifically, 21 were non-pianist musicians (10 males and 11 females, age range: 19 to 42 years old, mean age: 24.29 ± 5.02 years), 23 classical pianists (13 males and 10 females, age range: 18 to 34 years old, mean age: 24.83 ± 4.10 years), and 23 non-musicians (11 males and 12 females, age range, 21 to 35 years old; mean age: 25.86 ± 3.34 years).

In Table 1, we reported additional information about the musical training received by the non-pianist musicians and by the pianists involved in our study.

**Table 1:** Information about musical training received by non-pianist musicians and pianists recruited in the study. We reported the years of formal musical training and the years of daily practice with the musical instrument in four ranges of years. In the last column, “AC” refers to the age of commencement of the formal musical training, which is reported in terms of average ± standard deviation.

**Musicianship**	**Years of formal musical training**				**Years of musical instrument daily practice**				**AC**
	*0 to 2*	*3 to 5*	*6 to 9*	*10+*	*0 to 2*	*3 to 5*	*6 to 9*	*10+*	
Non-pianist musicians	0	2	10	12	0	1	9	14	8.41 ± 2.87
Pianists	0	3	5	15	0	2	5	16	7.57 ± 3.27

Participants had homogeneous socio-economic and educational backgrounds and signed the informed consent before the beginning of the experiment.

All the experimental procedures complied with the Declaration of Helsinki—Ethical Principles for Medical Research and were approved by the Ethics Committee of the Central Denmark Region (De Videnskabsetiske Komitéer for Region Midtjylland) (Ref 1-10-72-411-17).

### Experimental design and stimuli

As mentioned in the paragraph on the overview of the analysis pipeline, to study the brain dynamics of musical sequence recognition, we employed an old/new (Kayser et al. 2003) auditory sequence recognition task during MEG recording (Fig. 1a). First, participants were requested to listen to four repetitions of a MIDI version of the right-hand part of the entire prelude in C minor BWV 847 composed by J.S. Bach. The tones had the same duration, which was of approximately 250 ms. The full piece lasted about 2.5 min; thus, the total duration of the learning part was approximately 10 min (2.5 min repeated four times). Participants were asked to focus on the musical prelude and memorize it as much as possible. Second, they were presented with 80 short musical excerpts lasting 1,250 ms each and requested to indicate whether each excerpt belonged to the prelude by Bach (memorized musical sequence, “old,” 40 trials) or was a novel musical sequence (“new,” 40 trials). Subsequent analyses were performed on correctly recognized trials only. Importantly, the two categories of stimuli (memorized and novel musical sequences) were composed to be clearly distinguishable in the recognition task, even if they were matched among several variables, to prevent for potential confounds. Specifically, the two categories were matched for rhythm, volume, timbre, tempo, meter, tonality, information content ($IC$), and entropy ($H$). The memorized melodies consisted of excerpts of Bach’s prelude. We extracted one excerpt per musical bar, corresponding to the first five notes of the bar. These different excerpts were selected because they were representative of the melodic contour and of the general repetitive structure of Bach’s prelude. The novel musical sequences were created by assembling a series of tones with a melodic contour that was completely different from the one of Bach’s prelude excerpts. Importantly, such difference was present for all musical tones. By doing so, we designed a task that was challenging yet feasible, since the two categories of melodies presented several similarities, but were clearly different from one another. The 80 musical sequences are reported in musical notation in Figure SF1.

The $IC$ and $H$ were estimated for each tone of the prelude’s excerpts (mean $IC$: 5.70 ± 1.73, mean $H$: 4.70 ± 0.33) and of the novel melodies (mean $IC$: 5.92 ± 1.81, mean $H$: 4.78 ± 0.35) by using Information Dynamics of Music (IDyOM) (Pearce 2018). This robust method uses machine learning to return a value of $IC$ for the target note based on a combination of the preceding notes of the musical piece comprising the target note and of a set of rules learned from a large set of prototypical pieces of Western music. Thus, in our study, the $IC$ of each note of the musical sequences was computed using a model trained on both Bach’s prelude excerpts and the novel melodies (i) and on the large corpus of prototypical pieces of Western music usually employed by IDyOM (Pearce 2018) (ii). In this way, musical sequences of the two categories (memorized and novel sequences) with the same $IC$ were composed of a series of intervals and melodic contours that were quite similar and equally plausible in light of prototypical Western music.

Formally, the $IC$ represents the minimum number of bits required to encode ${e}_i$ and is described by equation (1):


(1) \begin{equation*} IC\left({e}_i|{e}_{\left(i-n\right)+1}^{i-1}\right)={\log}_2\frac{1}{p\left({e}_i|{e}_{\left(i-n\right)+1}^{i-1}\right)} \end{equation*}

where $ p\left({e}_i|{e}_{\left(i-n\right)+1}^{i-1}\right) $ is the probability of the event ${e}_i $ given a previous set of ${e}_{\left(i-n\right)+1}^{i-1} $ events.

The entropy gives a measure of the certainty/uncertainty of the upcoming event given the previous set of ${e}_{\left(i-n\right)+1}^{i-1}$ events and is calculated by equation (2):


(2) \begin{equation*} H\left({e}_{\left(i-n\right)+1}^{i-1}\right)=\sum_{e\in A}p\left({e}_i|{e}_{\left(i-n\right)+1}^{i-1}\right) IC\left({e}_i|{e}_{\left(i-n\right)+1}^{i-1}\right) \end{equation*}

Equation (2) shows that if the probability of a given event ${e}_i$ is 1, the probability of the other events in $A$ will be 0 and therefore, $H$ will be equal to 0 (maximum certainty). On the contrary, if all the events are equally likely, $H$ will be maximum (maximum uncertainty). Therefore, IDyOM returns an estimation of the predictability of each tone and uncertainty with which it can be predicted, coherently with the human perception (Sears et al. 2018).

The entire prelude and the musical excerpts were created by using Finale (MakeMusic, Boulder, CO) and then presented by adopting Presentation software (Neurobehavioural Systems, Berkeley, CA).

We collected structural images for each participant by employing magnetic resonance imaging (MRI), either on the same day as the functional MEG scan or on another day within one month.

### Data acquisition

We acquired both MRI and MEG data in two independent sessions. The MEG data were acquired by employing an Elekta Neuromag TRIUX system (Elekta Neuromag, Helsinki, Finland) equipped with 306 channels. The machine was positioned in a magnetically shielded room at Aarhus University Hospital, Denmark. Data were recorded at a sampling rate of 1000 Hz with an analogue filtering of 0.1 to 330 Hz. Prior to the measurements, we accommodated the sound volume at 50 dB above the minimum hearing threshold of each participant. Moreover, by utilizing a 3D digitizer (Polhemus Fastrak, Colchester, VT, USA), we registered the participants’ head shape and the position of four headcoils, with respect to three anatomical landmarks (nasion and left and right preauricular locations). This information was subsequently used to coregister the MEG data with the anatomical structure collected by the MRI scanner. The location of the headcoils was registered during the entire recording by using a continuous head position identification (cHPI), allowing us to track the exact head location within the MEG scanner at each time point. We utilized this data to perform an accurate movement correction at a later stage of data analysis. Finally, eyeblink and heart-beat activities were collected by applying two pairs of electrodes after cleaning the skin of the participants. To detect the eyeblink, one electrode was applied above and one below the right eye. To record the heart-beat activity one electrode was placed on the left last rib and the other one on the right collar bone of the participants.

The recorded MRI data corresponded to structural T1. The acquisition parameters for the scan were: voxel size = 1.0 × 1.0 × 1.0 mm (or 1.0 mm^3^); reconstructed matrix size 256 × 256; echo time (TE) of 2.96 ms and repetition time (TR) of 5,000 ms and a bandwidth of 240 Hz/Px. Each individual T1-weighted MRI scan was subsequently coregistered to the standard Montreal Neurological Institute (MNI) brain template through an affine transformation and then referenced to the MEG sensors space by using the Polhemus head shape data and the three fiducial points measured during the MEG session.

### Data preprocessing

The raw MEG sensor data (204 planar gradiometers and 102 magnetometers) was pre-processed by MaxFilter (Taulu and Simola 2006) for attenuating the interference originated outside the scalp by applying signal space separation (MaxFilter parameters: spatiotemporal signal space separation (SSS), movement compensation using cHPI coils (default step size: 10 ms); correlation limit between inner and outer subspaces used to reject overlapping intersecting inner/outer signals during spatiotemporal SSS: 0.98).

The data were converted into the Statistical Parametric Mapping (SPM) format and further analyzed in Matlab (MathWorks, Natick, Massachusetts, United States of America) using Oxford Centre for Human Brain Activity Software Library (OSL) (Woolrich et al. 2011), a freely available toolbox that combines in-house-built functions (https://github.com/leonardob92/LBPD-1.0.git) with existing tools from the FMRIB Software Library (FSL) (Woolrich et al. 2009), SPM (Penny et al. 2011), and Fieldtrip (Oostenveld et al. 2011). We applied a 48 to 52 Hz notch filter to correct for possible interference of the electric current and downsampled the data to 150 Hz. A few segments of the data (less than 0.5% of the whole dataset), contaminated by large artifacts, were removed after visual inspection. Then, to discard the interference of eyeblinks and heart-beat artifacts from the brain data, we performed independent component analysis (ICA) to decompose the original signal in independent components. We subsequently isolated and discarded the components that correlated with the time series recorded by the electrodes used for monitoring the eyeblink and heart-beat activities and rebuilt the signal from the remaining components (Mantini et al. 2011). In most cases, we rejected only two components, one for the eyeblink and one for the heart-beat activity. In a few cases, we rejected up to six components. This happened when more than one component picked up the eyeblink or the heart-beat activity. Finally, the data were low pass–filtered (40 Hz threshold) to improve the performance of the subsequently used decoding algorithm and univariate analyses and epoched in 80 trials (one for each musical excerpt) lasting 3,500 ms each (100 ms of prestimulus time). To be noted, when computing the static functional connectivity analysis we used data that were not low pass–filtered and therefore could be investigated in frequencies higher than 40 Hz. Then, correctly identified trials were analyzed by employing two different methodologies (multivariate pattern analysis and cluster based MCS of independent univariate analyses) to strengthen the reliability of the results as well as broaden the amount of information derived by the data.

In addition, it is worth noting that we replicated the key analyses reported in the main text of this manuscript using only minimal preprocessing steps. Specifically, the analyses included decoding, MCS on MEG sensor data, and MCS on source reconstructed data. The preprocessing steps consisted of applying MaxFilter and ICA to remove eye-blink and heartbeat artifacts. The results of these analyses, which are reported in the Supplementary Materials, show that not performing low-pass and notch filters and using a different sampling rate (250 Hz) did not affect the significance of our original findings (see Figures SF2 and SF3 for a comparison between the results obtained following the two preprocessing pipelines).

### Multivariate pattern analysis

We conducted a multivariate pattern analysis to decode different neural activity associated with the recognition of previously memorized versus novel musical sequences. Specifically, we employed support vector machines (SVMs) (Cichy et al. 2014), analyzing each participant independently. MEG data were arranged in a 3D matrix (channels × time points × trials) and submitted to the supervised learning algorithm. To avoid overfitting, we employed a leave-one-out cross-validation approach to train the SVM classifier to decode the two conditions. This procedure consisted of dividing the trials into *N* different groups (here *n* = 8) and, for each time point, assigning *N − 1* groups to the training set and the remaining *Nth* group to the testing set. Then, the performance of the classifier to separate the two conditions was evaluated. This process was carried out 100 times with random reassignment of the data to training and testing sets. Finally, the decoding accuracy time series were averaged together to obtain a final time series reflecting the performance of the classifier for each participant.

To identify the channels that were carrying the highest amount of information required for decoding the two experimental conditions, we followed the procedure described by Haufe and colleagues (Haufe et al. 2014) and computed the decoding sequences from the weights returned by the SVM. Moreover, we computed the confusion matrix for each time point, obtaining the four time series reported in Figure SF4.

Finally, to assess whether the two experimental conditions were differentiated by neural sequences stable over time, we performed a temporal generalization multivariate analysis. The algorithm was the same as the one described above, with the difference that in this case we used each time point of the training set to predict not only the same time point in the testing set but also all time points (Cichy et al. 2014; King and Dehaene 2014).

In both cases, to test whether the decoding results were significantly different from the chance level (50%), we used a sign permutation test against the chance level for each time point and then corrected for multiple comparisons by applying FDR correction (α = 0.05; FDR-adjusted *q* < 0.026 for non-temporal generalization results and α = 0.02; FDR-adjusted *q* < 0.005 for temporal generalization results).

### Univariate tests and Monte Carlo simulations

The multivariate pattern analysis is a powerful tool that requires relatively few preprocessing steps for returning an estimation of the different neural activity associated with two or more experimental conditions. However, this technique does not identify which condition is stronger than the other nor the polarity of the neural signal characterizing the experimental conditions. To answer these questions and strengthen our results, we employed a different approach by calculating several univariate *t*-tests and then correcting for multiple comparisons by using MCS.

Before computing the *t*-tests, in accordance with many other MEG and electroencephalography (EEG) task studies (Gross et al. 2013), we averaged over trials in each condition, obtaining two mean trials, one for the memorized and one for the novel musical sequences. Then, we combined each pair of planar gradiometers by sum-root square. Afterward, we computed a *t*-test for each MEG channel and each time point in the time range 0 to 2.500 s, contrasting the two experimental conditions. Independently for the two MEG sensor categories, we reshaped the matrix for obtaining, for each time point, a 2D approximation of the MEG channels layout that we binarized according to the *P*-values obtained from the previous *t*-tests (threshold = 0.01) and the sign of *t*-values. The resulting 3D matrix (*M*, 2D × time) consisted of 0 s when the *t*-test was not significant and 1 s when it was. Then, to correct for the multiple comparisons happening in these univariate analyses, we identified the clusters of 1 s and assessed their significance by running MCS. Specifically, we made 1000 permutations of the elements of the original binary matrix *M*, identified the maximum cluster size of 1 s, and built the distribution of the 1,000 maximum cluster sizes. Finally, we considered significant the original clusters that had a size bigger than the 99.9% maximum cluster sizes of the permuted data. The whole MCS procedure was performed for gradiometers and magnetometers (in the significant time-window emerged from gradiometers, see SI Appendix SR1 for details), for memorized versus novel musical sequences and vice versa.

### Relationship between neural activity and behavioral measures

We investigated whether the neural activity underlying the musical recognition task was modulated by musical expertise and individual differences along the following four behavioral measures: WM skills (i), esthetical judgment of the musical piece used in the study (ii), previous familiarity with Bach’s prelude (iii), and the Goldsmith Musical Sophistication Index (GOLD-MSI) (iv) (Müllensiefen et al. 2013), which measures the ability of engaging with music. Regarding WM, we employed the widely used Wechsler Adult Intelligence Scale (WAIS-IV) (Wechsler 1997), which returned a reliable measure of individual WM. With regard to esthetical judgment of Bach’s prelude, we utilized a 7-score Likert scale from −3 (very unpleasant) to +3 (very pleasant). Previous familiarity with Bach’s prelude was assessed asking participants to mark the number corresponding to one of the following options: *1) I have never heard it before; 2) I have occasionally heard it; 3) I sometimes listen to it; 4) I usually listen to it; 5) I played it for myself; and 6) I played it in front of a public*. Further, in our sample, only six participants declared to have previously played the Bach’s prelude that we used in the study in front of a public and only four of them stated that they still remembered a few bars of it.

Then, we used the averaged brain activity over the significant gradiometer channels returned by our previous analysis. Here, we computed the difference between the neural activity underlying recognition of memorized versus novel musical sequences. Then, we computed independent Pearson’s correlations for each time point of the resulting time series and the four behavioral measures described above [WM skills (i), esthetical judgment of Bach’s prelude used in the study (ii), previous familiarity with Bach’s prelude (iii), and the GOLD-MSI (iv)]. Moreover, we contrasted the brain activity underlying music recognition across pianists, non-pianist musicians, and nonmusicians. For this analysis, we used one analysis of variance (ANOVA) for each time point.

The time series obtained from the correlations and the ANOVA were binarized according to the outcome of the tests [one was assigned to the significant tests (*P* < 0.05), while zero to the nonsignificant ones]. Those values were submitted to a 1D MCS with an α level = 0.001, to correct for multiple comparisons. First, we extracted the clusters of the significant results emerged from the correlations (here, cluster means group of contiguous significant values [ones]). Then, we computed 1,000 permutations and for each of them, we randomized the order of the binarized values and computed the maximum cluster size of the detected clusters of significant values. Finally, we built a reference distribution of the 1,000 maximum cluster sizes (obtained from the 1,000 permutations) and considered significant the original clusters that were larger than the 99.9% of the permuted ones.

### Source reconstruction

#### Beamforming

The brain activity collected on the scalp by MEG channels was reconstructed in source space. First, we coregistered the individual MRI scan with the corresponding 3D coordinates recorded during the MEG session and transformed the native space into MNI space. In the few cases (three) when the individual MRI scan was not available, we used a brain template (MNI 152 T1 template). Second, using OSL (Woolrich et al. 2011), we applied a local-spheres forward model and a beamformer approach as the inverse method (Hillebrand and Barnes 2005) (Fig. 1b and c). We utilized an 8 mm grid and both magnetometers and (noncombined) planar gradiometers. We accounted for the differing signal strength of magnetometers and gradiometers by converting their values in standardized *z*-scores. The spheres model used as forward model depicted the MNI-coregistered anatomy as a simplified geometric model, fitting a sphere separately for each sensor (Hillebrand and Barnes 2005). The beamforming that we employed as inverse method utilized a diverse set of weights sequentially applied to the source locations for isolating the contribution of each source to the activity recorded by the MEG channels for each time point (Hillebrand and Barnes 2005; Brookes et al. 2007). The covariance matrix necessary to compute those weights was calculated on the matrix obtained by concatenating the data of all trials for the two conditions and normalized according to Luckhoo et al. (2014) for counterbalancing the reconstruction bias toward the center of the head.

#### General linear model

An independent general linear model (GLM) was calculated sequentially for each time point at each dipole location and for each experimental condition (Hunt et al. 2012). At first, reconstructed data were tested against its own baseline to calculate the statistics of neural sources of the two conditions memorized and novel musical sequences. Then, after computing the absolute value of the reconstructed time series to avoid sign ambiguity of the neural signal, first-level analysis was conducted, calculating contrast of parameter estimates (memorized versus novel musical sequences) for each dipole and each time point. Those results were submitted to a second-level analysis, using one-sample *t*-tests with spatially smoothed variance obtained with a Gaussian kernel (full-width at half-maximum: 50 mm).

Then, to correct for multiple comparisons, a cluster-based permutation test (Hunt et al. 2012) with 5,000 permutations was computed on second-level analysis results, taking into account the significant time range emerged from the MEG sensors MCS significant gradiometer cluster. Therefore, we performed one permutation test on source space, using an α level of 0.05, corresponding to a cluster forming threshold of *t* = 1.7.

#### Brain activity underlying musical sequences development

Then, as depicted in Fig. 1d, we performed an additional analysis considering the brain activity underlying the processing of each tone forming the musical sequences. To do that, we computed a GLM for each time point and source location. Then, we averaged over the time points forming each of the five time windows associated with the duration of the musical tones (0 to 250 ms, 251 to 500 ms, 501 to 750 ms, 751 to 1,000 ms, 1,001 to 1,250 ms). Finally, we corrected for multiple comparisons with a cluster-based permutation test, as described above (Hunt et al. 2012). Here, when computing the significant clusters of brain activation independently for the two experimental conditions (memorized and novel musical sequences), we computed 10 permutation tests on source space, adjusting the α level to 0.005 (0.05/10), corresponding to a cluster forming threshold of *t* = 2.7. Regarding memorized versus novel musical sequences, we performed five tests and therefore, the α level became 0.01 (0.05/5), corresponding to a cluster forming threshold of *t* = 2.3.

### Functional connectivity preprocessing

After reconstructing the data into source space, we constrained the beamforming results into the 90 noncerebellar regions of the automated anatomic labelling (AAL) parcellation, a widely used and freely available template (Tzourio-Mazoyer et al. 2002) in line with previous MEG studies (Brookes et al. 2016) and corrected for source leakage (Colclough et al. 2015). Finally, since we were interested in studying the functional connectivity of evoked responses, according to a large number of MEG and EEG task studies (Gross et al. 2013), we averaged the trials over conditions, obtaining two mean trials, one for memorized and one for novel musical sequences. In order to minimize the probability of analyzing trials that were correctly recognized by chance, here, we only considered the 20 fastest (mean RT: 1,770 ± 352 ms) correctly recognized previously memorized musical sequences (mean RT: 1,717 ± 381 ms) and novel musical sequences (mean RT: 1,822 ± 323 ms) excerpts. The same operation has been carried out for the resting state that served as baseline. Here, we created 80 pseudo-trials with the same length of the real ones, starting at random time points of the recorded resting-state data.

This procedure has been carried out for five different frequency bands (0.1 to 2 Hz, 2 to 8 Hz, 8 to 12 Hz, 12 to 32 Hz, 32 to 75 Hz) (Lee et al. 2018).

### Static functional connectivity and degree centrality

We estimated the static functional connectivity (SFC) calculating Pearson’s correlations between the envelope (computed using the Hilbert transform; Liu 2012) of each pair of brain areas time courses. This procedure has been carried out for both task and baseline and for each of the five frequency bands considered in the study. Afterward, we averaged the connectivity matrices in order to obtain one global value of connectivity for each participant and each frequency band. These values were submitted to an ANOVA to discover which frequency band yielded the strongest connectivity effects (Allen et al. 2019). A follow up post-hoc analysis was conducted using Tukey’s correction for multiple comparisons. Then, for all frequency bands, we computed Wilcoxon sign-rank tests comparing each pair of brain areas for recognition task versus baseline, aiming to identify the functional connectivity specifically associated with the task. To assess the resulting connectivity matrix *B*, we identified the degree of each region of interest (ROI) and tested its significance through MCS^59^.

In graph theory the weighted degree of each vertex *v* (here each brain area) of the graph *G* (here the matrix *B*) is given by the sum of the connection strengths of *v* with the other vertexes of *G*, showing the centrality of each *v* in *G* (Rubinov and Sporns 2010). We computed the degree of each vertex of *B* for each musical tone, obtaining a 90 × 1 vector (${s}_t$). Then, through MCS, we assessed whether the vertices of *B* had a significantly higher degree than the degrees obtained permuting the elements of *B*. Specifically, we made 1,000 permutations of the elements in the upper triangle of *B* and we calculated a 90 × 1 vector ${d}_{v,p}$ containing the degree of each vertex *v* for each permutation *p*. Combining vectors ${d}_{v,p}$ we obtained the distribution of the degrees calculated for each permutation. Finally, we considered significant the original degrees stored in ${s}_t$ that randomly occurred during the 1,000 permutations less than two times. This threshold was obtained by dividing the α level (0.001) by the five frequency bands considered in the study. The α level was set to 0.001 since this is the threshold that, during simulations with input matrices of uniformly distributed random numbers, provided no false positives. This procedure was carried out for each frequency band and for both experimental conditions.

## Results

### MEG sensor data

Our first analysis was conducted on MEG sensor data and focused on the brain activity underlying the recognition of the previously memorized versus novel musical sequences. On average, participants correctly identified the 78.15 ± 13.56% of the previously memorized melodies [mean *d’* score = 1.70 ± 1.03; mean reaction times (RTs): 1,871 ± 209 ms] and the 81.43 ± 14.12% of the novel melodies (mean *d’* score = 2.38 ± 1.63; mean RTs: 1915 ± 135 ms). Subsequent MEG sensor data analyses were conducted on correct trials only.

#### Multivariate pattern analysis

**Fig. 2:**
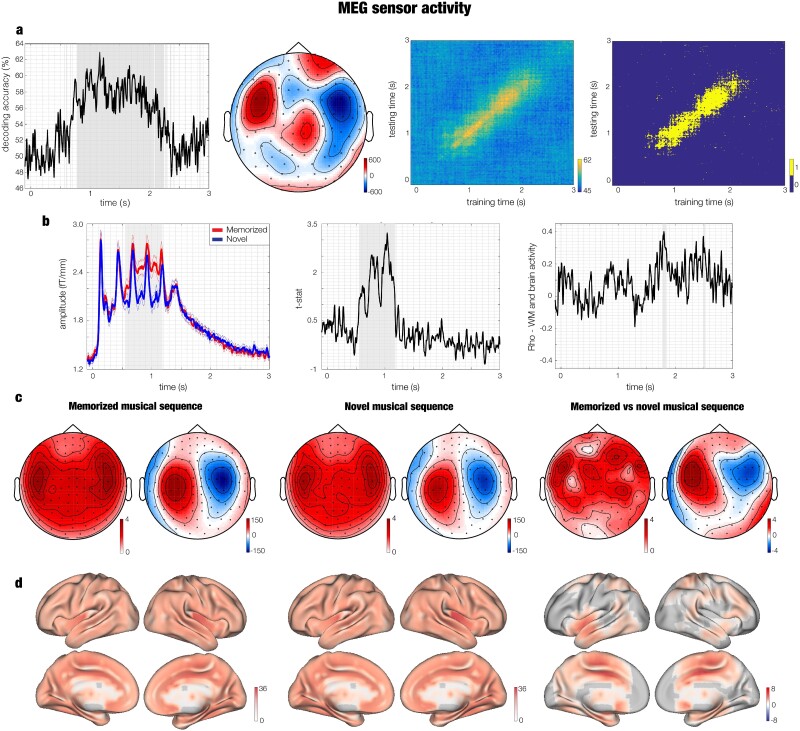
Brain activity underlying memorized versus novel musical sequences. a) Multivariate pattern analysis decoding the different neural activity associated with memorized versus novel musical sequences. Decoding time series (left), spatial sequences depicted as topoplot (middle left), temporal generalization decoding accuracy (middle right), and statistical output of significant prediction of training time on testing time (right). b) The left plot shows the amplitude associated with memorized (red) and novel musical sequences (blue). The middle plot illustrates the *t*-statistics related to the contrast between memorized versus novel musical sequences. The right plot shows the correlation between WM abilities and the neural responses underlying recognition of the memorized versus novel musical sequences. Thinner lines depict standard errors. The plots refer to the average over the gradiometer channels forming the significant cluster outputted by MEG sensor MCS. c) Three couples of topoplots showing brain activity for gradiometers (left of each pair, fT/cm) and magnetometers (right of each pair, fT) within the significant time window emerged from MCS. First couple of topoplots depicts the neural activity underlying the recognition of the previously memorized musical sequences, second couple refers to the novel musical sequences, while the third one represents the statistics (*t*-values) contrasting the brain activity underlying recognition of memorized versus novel musical sequences. d) Neural sources for the recognition of memorized sequences (left), novel sequences (middle), and their contrast (right). The values are *t*-statistics.

As depicted in Fig. 2a, we conducted a multivariate pattern analysis using a support vector machine (SVM) classifier (see details in the Materials and Methods section) to decode different neural activity associated with the recognition of previously memorized versus novel musical sequences. This analysis resulted in a decoding time series showing how neural activity differentiated the two experimental conditions. The decoding time series was significantly different from chance level in the time range 0.8 to 2.1 s from the onset of the first tone (*q* < 0.026, false-discovery rate (FDR)–corrected, Fig. 2a, top left). The highest accuracy was reached when predicting the neural activity underlying recognition of novel melodies, while the prediction of previously memorized melodies was less accurate, as shown by confusion matrix computed for each time point (Figure SF4).

To evaluate the persistence of discriminable information over time, we applied a temporal generalization approach by training the SVM classifier at a given time point *t*, as before, but testing across all other time points. FDR-corrected (*q* < 0.005) results are depicted in Fig. 2a (top right) showing that performance of the classifier was significantly above chance even a few hundreds of milliseconds beyond the diagonal.

In addition, we computed the same analysis after following only minimal preprocessing steps (i.e. MaxFilter and ICA for removing eyeblink and heart-beat artifacts) and using a sampling rate of 250 Hz. The analysis returned very similar results, although overall less strong. Indeed, the decoding time series was significantly different from chance level in several time windows in the time range 0.8 to 2.1 s from the onset of the first tone (*q* < 0.026, FDR-corrected). Moreover, temporal generalization analysis confirmed that the performance of the classifier was significantly above chance even a few hundreds of milliseconds beyond the diagonal (*P* < 0.005). A graphical depiction of the comparison between the two analyses that differed only for the preprocessing steps and for the sampling rate is provided in Figure SF2. Our procedure clearly shows that the significance of the results of our main analysis pipeline was not affected by computing low-pass and notch filter and by using 150 Hz instead of a different sampling rate such as 250 Hz.

#### Univariate tests and MCSs

First, we contrasted the previously memorized versus novel musical sequences (*t*-test threshold = 0.01, MCS threshold = 0.001, 1000 permutations), considering the positive *t*-values only (which is when the memorized music was associated with a stronger brain activity than the novel melodies). We performed this analysis in the time range 0 to 2.5 s by using combined planar gradiometers only. This procedure yielded the identification of one main significant cluster (MCS *P* < 0.001; time: 0.547–1.180 s, size: 2117), as depicted in Fig. 2b and c and reported in detail in Tables ST1 and ST3. In addition, we computed the same analysis after following only minimal preprocessing steps (i.e. MaxFilter and ICA for removing eyeblink and heart-beat artifacts) and using a sampling rate of 250 Hz. The results returned one large cluster that was very similar to the one described above (MCS *P* < 0.001; time: 0.640 to 1.140 s, size: 2,649). Detailed statistics is reported in Table ST3, while a graphical depiction of the comparison between the two analyses that differed only for the preprocessing steps and for the sampling rate is provided in Figure SF2. Our procedure clearly shows that the results of our main analysis pipeline were not affected by computing low-pass and notch filter and by using 150 Hz instead of a different sampling rate such as 250 Hz.

After working with the gradiometers, we computed analyses for the magnetometers. Here, based on the significant cluster appearing, we computed the same algorithm one more time for magnetometers only, within the significant time range emerged for the first MCS (0.547 to 1.180 s, *P* < 0.001, Table ST1). This two-step procedure was necessitated by the sign ambiguity typical of magnetometer data and returned three significant clusters (positive magnetometers: MCS *P* < 0.001; time: 0.627 to 1.180 s, size: 817: negative magnetometers: Cluster I—MCS *P* < 0.001; time: 0.727 to 0.880 s, size: 190; Cluster II—MCS *P* < 0.001; time: 0.960 to 1.133 s, size: 168).

Then, the same procedure was carried out by considering the results where the brain activity associated with the novel melodies exceeded the one elicited by Bach’s prelude excerpts. This analysis returned eight small significant clusters (size range: 6 to 14, *P* < 0.001) shown in Table ST2.

#### Relationship between brain activity and behavioral measures

Once we established that the recognition of the memorized and novel musical sequences gave rise to clearly different brain activity, we investigated whether such activity was modulated by individual differences such as WM and musical skills related to the Bach’s prelude used in the study.

We correlated each time point of the brain activity time series with four behavioral measures: WM skill (i), esthetical judgment of the Bach’s prelude (ii), previous familiarity with the Bach’s prelude (iii), and the GOLD-MSI (iv), which measures the ability of engaging with music. We corrected for multiple comparisons by using MCS with significance level α = 0.001. Overall, the results showed that the neural activity was not correlated to such measures. Indeed, detailed analysis revealed only few small significant clusters. Two clusters at the gradiometer level were found for WM (Cluster I: MCS *P* < 0.001; time: 1.77 to 1.85, mean *r* = 0.32; Cluster II: MCS *P* < 0.001; time: 2.48 to 2.53, mean *r* = 0.30, Fig. 2b), meaning that participants with higher WM presented a stronger neural activity underlying musical recognition. Regarding liking, we detected two significant clusters (Cluster I: MCS *P* < 0.001; time: 2.40 to 2.45, mean *r* = 0.33 (Figure SF5); Cluster II: MCS *P* < 0.001; time: 1.11 to 1.14, mean *r* = −0.27). The two clusters were small and pointed to different directions of the correlation with the neural data (one was positive and the other negative), suggesting that the esthetical judgment was not a good predictor of the brain activity. Additionally, both familiarity with Bach’s prelude and the GOLD-MSI returned only one small cluster showing a negative correlation: Cluster I: MCS *P* < 0.001; time: 1.18 to 1.21, mean *r* = −0.28. (familiarity); Cluster I: MCS *P* < 0.001; time: 0.10 to 0.13, mean *r* = −0.29 (GOLD-MSI) (Figure SF5). Finally, no significant differences were detected when contrasting the brain activity underlying music recognition across pianists, non-pianist musicians, and nonmusicians (Figure SF5).

### Source-reconstructed data

To identify the neural sources of the signal, we employed a beamforming approach and computed a GLM for assessing, at each time point, the independent neural activity associated with the two conditions as well as their contrasts.

#### Main cluster of previously memorized versus novel musical sequences

We identified the neural sources of the gradiometers significant cluster emerging from the MEG sensor data when contrasting memorized versus novel musical sequences. Here, we performed one permutation test in source space, with an α level of 0.05, which, in our case, corresponded to a cluster forming threshold of *t* = 1.7. As depicted in Fig. 2d, results showed a strong activity originating in the primary auditory cortex, insula, hippocampus, frontal operculum, cingulate cortex, and basal ganglia. Detailed statistics are provided in Table ST4. In addition, we computed the same analysis after following only minimal preprocessing steps (i.e. MaxFilter and ICA for removing eyeblink and heart-beat artifacts) and using a sampling rate of 250 Hz. The analyses returned extremely similar results to the ones described above, localizing the significant difference between memorized and novel musical sequences auditory cortex, insula, hippocampus, frontal operculum, cingulate cortex, and basal ganglia. Detailed statistics is reported in Table ST4, while a graphical depiction of the comparison between the two analyses that differed only for the preprocessing steps and for the sampling rate is provided in Figure SF2. Our procedure clearly shows that the results of our main analysis pipeline were not affected by computing low-pass and notch filter and by using 150 Hz instead of a different sampling rate such as 250 Hz.

#### Dynamic brain activity during development of musical sequences

**Fig. 3:**
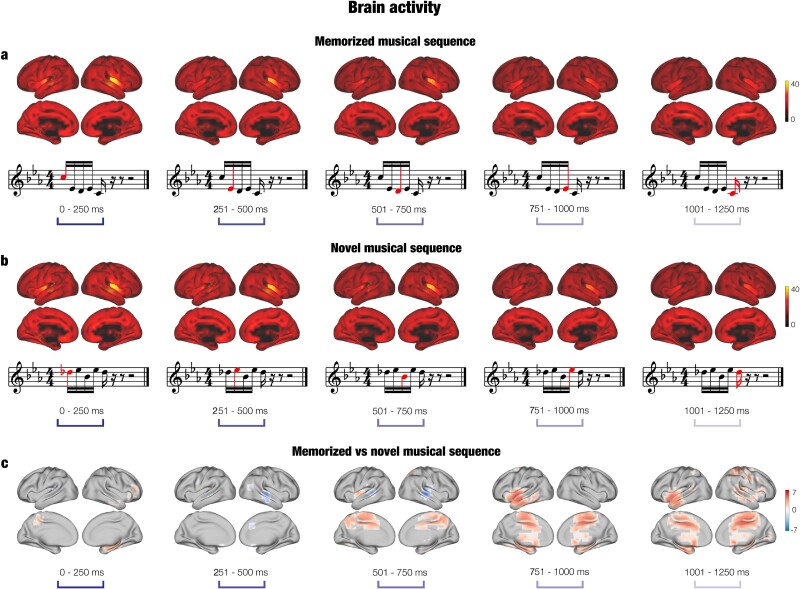
Brain activity over time. a) Brain activity (localized with beamforming) associated with the recognition of previously memorized musical sequences (top row). Such sequences were extracted from the Bach’s prelude that participants attentively listened to before doing the recognition task. The bottom row depicts an example trial for the memorized sequences. Red tones illustrate the dynamics of the musical excerpt. b) Brain activity underlying the detection of the novel musical sequence (top row) and musical representation of one example trial (bottom row). c) Contrast (*t*-values) over time between the brain activity underlying memorized versus novel musical sequences.

To reveal the specific brain activity dynamics underlying the recognition of the musical sequences, we carried out a further analysis for each musical tone forming the musical sequence. Here, we adopted a stricter cluster forming threshold of *t* = 2.7 (see Materials and Methods for details). As depicted in Fig. 3a and b, we found significant activity within primary auditory cortex and insula, especially in the right hemisphere, for both experimental conditions. This activity decreased over time, following the unfolding of the musical sequences. Conversely, the contrast between memorized versus novel music gave rise to a burst of activity for the memorized Bach’s excerpts increasing over time, especially with regard to the last three tones of the musical sequences, as shown in Fig. 3c. This activity was mainly localized within hippocampus, frontal operculum, cingulate cortex, insula, inferior temporal cortex, and basal ganglia. We report detailed clusters statistics in Table ST5. In addition, we computed the same analysis after following only minimal preprocessing steps (i.e. MaxFilter and ICA for removing eyeblink and heart-beat artifacts) and using a sampling rate of 250 Hz. The analyses returned extremely similar results to the ones described above, localizing the significant difference between memorized and novel musical sequences auditory cortex, insula, hippocampus, frontal operculum, cingulate cortex, and basal ganglia. Moreover, the strongest difference between the two conditions was obtained for the last three tones of the musical sequences. Detailed statistics is reported in Table ST5, while a graphical depiction of the comparison between the two analyses that differed only for the preprocessing steps and for the sampling rate is provided in Figure SF3. Our procedure clearly shows that the results of our main analysis pipeline were not affected by computing low-pass and notch filter and by using 150 Hz instead of a different sampling rate such as 250 Hz.

### Static functional connectivity

To obtain a better understanding of the brain dynamics underlying recognition, we complemented our brain activity results with an investigation of the static functional connectivity of the evoked responses.

**Fig. 4:**
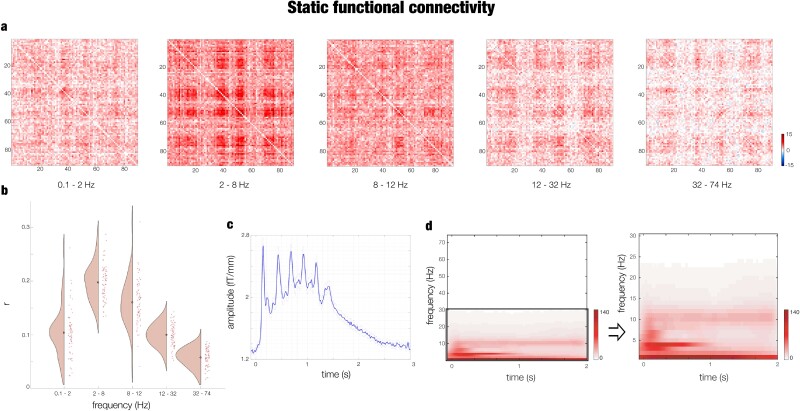
Static functional connectivity. a) Contrast between recognition task (memorized and novel musical sequences averaged together) and baseline SFC matrices calculated for five frequency bands: 0.1 to 2 Hz, 2 to 8 Hz, 8 to 12 Hz, 12 to 32 Hz, and 32 to 74 Hz. b) Violin-scatter plot showing the average of the SFC matrices over their two dimensions for all participants. c) Averaged MEG gradiometer channels waveform of the brain activity associated with the recognition task. d) Power spectra for the evoked responses associated with the recognition task computed for all MEG channels. The first power spectra matrix reflects the analysis from 1 to 74 Hz in 1 Hz intervals, while the second reflects the analysis from 1 to 30 Hz in 1 Hz intervals.

After constraining the MEG preprocessed data to the 90 noncerebellar parcels of the AAL parcellation, we estimated static functional connectivity by using Pearson’s correlations in five frequency bands: 0.1 to 2 Hz, 2 to 8 Hz, 8 to 12 Hz, 12 to 32 Hz, 32 to 74 Hz. Then, we tested the overall connectivity strengths of the five frequency bands during auditory recognition by employing ANOVA. The test was significant [*F*(4,330) = 187.02, *P* < 1.0e-07). As depicted in Fig. 4a and b, post-hoc analysis highlighted especially that the 2 to 8 Hz band had a stronger connectivity profile than all other frequency bands (*P* < 1.0e-07).

To detect the significance of each brain region centrality within the whole-brain network for the auditory recognition task, we contrasted the brain connectivity matrices associated with the task versus baseline by performing a Wilcoxon signed-rank test for each pair of brain areas. Then, the resulting *z*-values matrix was submitted to a degree MCS (see Materials and Methods for details). We computed this analysis independently for the five frequency bands, and therefore, we considered significant the brain regions whose *P*-value was lower than the α level divided by 5 (2.0e-04). The results for 2 to 8 Hz are depicted in Figure SF6 and reported as follows: left Rolandic operculum (*P* < 1.0e-07), insula (*P* < 1.0e-07), hippocampus (*P* = 5.5e-05), putamen (*P* < 1.0e-07), pallidum (*P* < 1.0e-07), caudate (*P* = 1.1e-05), thalamus (*P* < 1.0e-07), Heschl’s gyrus (*P* < 1.0e-07), superior temporal gyrus (*P* < 1.0e-07), right superior temporal gyrus (*P* = 1.1e-06), Heschl’s gyrus (*P* < 1.0e-07), thalamus (*P* < 1.0e-07), parahippocampal gyrus (*P* = 4.3e-05), pallidum (*P* < 1.0e-07), putamen (*P* < 1.0e-07), amygdala (*P* < 1.0e-07), insula (*P* < 1.0e-07), and Rolandic operculum (*P* < 1.0e-07). Additional results related to the other frequency bands are reported in supporting information (SI) Appendix (SR2).

Conversely, the degree MCS of the contrasts between memorized versus novel melodies yielded no significant results.

## Discussion

In this study, we detected the spatiotemporal dynamics of the whole-brain activity and functional connectivity during recognition of previously memorized auditory sequences compared to matched novel melodies.

First, by using a broadband multivariate pattern analysis and MCS of massive univariate data, we found converging evidence that the brain activity elicited by the recognition of musical excerpts extracted from Bach’s prelude compared to the novel musical sequences gave rise to significant changes in widespread regions including the primary auditory cortex, superior temporal gyrus, cingulate gyrus, hippocampus, basal ganglia, insula, and frontal operculum. Notably, the neural difference reflecting the recognition of memorized versus novel musical sequences extended from approximately 700 to 2,000 ms after the onset of the first tone of the melodies. This suggests that the brain discriminated the two categories of musical sequences, especially from tone number three of the sequences. Interestingly, this difference in neural activity extended up to 2,000 ms, which corresponded to about 100 ms after participants categorized the musical sequences by using the response pad (the mean reaction time was approximately 1,900 ms for both categories of sequences). This could indicate that the elaborated process of recognition and discrimination of the two categories of musical sequences requires a widespread network of brain areas whose activity was differentiated for more than 1,000 ms.

Second, we inspected this finding further by estimating static functional brain connectivity evolving over time. Here, the recognition of both previously memorized and novel auditory sequences was accompanied by significant centrality within the whole-brain network of several brain regions including the insula, hippocampus, cingulate gyrus, auditory cortex, basal ganglia, frontal operculum, and subgenual and orbitofrontal cortices. This result emerged only for the frequency band: 2 to 8 Hz.

First of all, the results presented in the current study either replicate or closely align to findings reported in our prior research on the long-term encoding and recognition of music (Bonetti et al. 2021a; Bonetti et al. 2022a; Bonetti et al. 2024; Fernandez-Rubio et al. 2022; Fernández-Rubio et al. 2022), as well as with several studies investigating music, auditory perception, and memory processes. For instance, the brain activity observed in our study during the recognition of musical sequences aligns with previous research indicating auditory processes associated with the primary auditory cortex and insula (Mutschler et al. 2007; Brattico and Pearce 2013). Moreover, in this study we observed stronger activity underlying the recognition of the memorized sequence in brain areas related to memory recognition such as hippocampus, medial temporal cortices (Brown and Aggleton 2001; Bird 2017), and cingulate cortices (Teixeira et al. 2006). Additionally, the recognition of excerpts from Bach’s prelude was associated with a stronger activity of brain regions previously related to evaluative processes (Bach et al. 2008; Stephenson-Jones et al. 2016) and pleasure (Kringelbach 2010) such as the cingulate gyrus and subgenual cortices, as well as parts of the basal ganglia. Finally, recognition of memorized music was accompanied by stronger activity in brain regions responsible for fine-grained auditory elaboration and prediction error such as the inferior temporal cortex (Zatorre et al. 2002) and insula (Limongi et al. 2013). Of particular interest is the involvement of the hippocampus and cingulate gyrus, whose role in auditory processing is not completely clear. The hippocampus has been previously reported in relation to music, especially in fMRI studies that investigated memory processing for sounds (Baumgartner et al. 2006; Alluri et al. 2015). Nevertheless, less is known about its the fast temporal dynamics in relation to music processing. Interestingly, our results indicated that the hippocampus was mainly relevant for the abstract recognition of the melodic sequence, while its involvement in the auditory processing of single sounds was reduced. The cingulate gyrus is an associative area that has been connected to several cognitive processes involving music imagery, but not memory (Criscuolo et al. 2019; Criscuolo et al. 2022). Notably, in this study, we showed that it was strongly involved in music recognition, highlighting its relevance for auditory memory. Future research should further investigate the specific role played by the cingulate gyrus, clarifying, for example, whether it is mainly related to the actual recognition process or if it modulates the attentional resources for the task. Interestingly, several brain regions we identified, such as the orbitofrontal cortex, cingulate gyrus, insula, and thalamus, are part of the limbic system. Despite our stimuli being brief musical excerpts not designed to elicit a wide range of emotional responses, these regions were still activated. Although definitive conclusions cannot yet be drawn, this activation may be due to two factors. First, the identified limbic regions are implicated in diverse functions beyond emotions (Bach et al. 2008; Limongi et al. 2013; Stephenson-Jones et al. 2016; Criscuolo et al. 2019; Criscuolo et al. 2022) and, in this context, they may be more engaged in evaluative and memory functions rather than emotional ones. Alternatively, participants may have experienced a sense of recognition and correctness when identifying the musical excerpts as familiar melodies, which could evoke an emotional response, arguably of pleasure, thereby engaging the limbic system. Future research should specifically investigate these possibilities.

After verifying that our results were consistent with and replicated findings from our prior research and other studies in the field, we proceeded to test our novel hypotheses. The first hypothesis was to examine both the commonalities and distinctions in brain activity and functional connectivity during the recognition of musical sequences. Our connectivity analysis revealed both important similarities and differences with the brain activity, coherently with our previous works on the topic (Bonetti et al. 2022a; Fernandez-Rubio et al. 2022; Fernández-Rubio et al. 2022). A key similarity consists of the significant brain areas emerged by conducting activity and functional connectivity analyses. Indeed, both analyses returned a network comprising insula, hippocampus, cingulate gyrus, auditory cortex, basal ganglia, frontal operculum, and subgenual and orbitofrontal cortices. This suggests that processing of auditory sequences does not only require the mere activation of a large network of brain areas but also their communication over time. Interestingly, a relevant difference between activity and functional connectivity was that the primary auditory cortex did not play a crucial role in functional connectivity, while it was central in the brain activity elicited by the presentation of the sounds. Moreover, the main connectivity patterns emerged for brain regions not only related to auditory processing but also to higher sound and linguistic elaborations such as the insula, inferior temporal cortex (Zatorre et al. 2002; Limongi et al. 2013), and frontal operculum (Koelsch et al. 2006). Furthermore, we also observed other central brain areas that have been previously related to evaluative (e.g. orbitofrontal and subgenual cortices; Bach et al. 2008) and memory recognition (e.g. hippocampus; Squire and Bayley 2007 and basal ganglia; Stephenson-Jones et al. 2016) processes. Another relevant difference was that while the activity of the brain areas was strongly diverse in the recognition of the previously memorized versus novel sequences, the functional connectivity analysis did not reveal any difference between the two experimental conditions. This finding suggests that the communication between the large network of brain areas that we revealed may be necessary to process auditory sequences, while the key for discerning previously memorized from novel melodies may be in the differential activity over time of some of the key brain regions comprised in the large network. Lastly, it is important to emphasize that we observed significant connectivity patterns only within the 2 to 8 Hz frequency range. Given that our study presented sounds at a frequency of 4 Hz (with each sound lasting 250 ms), we cannot definitively determine whether the heightened connectivity in the 2 to 8 Hz range reflects a specific brain rhythm or a stimulus-driven brain response. Future studies are needed to explore this observation further. Moreover, future research should also investigate this phenomenon under different experimental conditions, including not only comparing resting state to active listening during memory tasks but also incorporating passive listening conditions. These efforts will help to refine our understanding of the functional connectivity mechanisms involved in music recognition.

The second hypothesis consisted of comparing the brain networks revealed by the functional connectivity analysis with those obtained using the same analysis for sound encoding in Bonetti et al. (2021b). This comparison holds particular significance because our study utilized a different dataset from the same participants as Bonetti et al. (2021b), enabling direct and valuable insights into similarities and differences. Remarkably, the network of brain areas emerging for the recognition of musical sequences highly resembled the one reported in our previous work. Indeed, primary and secondary auditory cortex, insula, hippocampus, basal ganglia, cingulate gyrus, and frontal operculum were highly involved both in encoding and recognition of sounds and sequences. Interestingly, while encoding mainly recruited a network of brain areas in the right hemisphere (both in terms of activity and connectivity), recognition of musical sequences was associated with activity and centrality of both hemispheres, with an overall stronger involvement of the left one. Taken together, our two studies reinforce the thesis that for both encoding and recognition of music a large network of functionally connected brain regions typically involved in auditory, memory and evaluative processes is required.

The third hypothesis involved employing temporal generalization in multivariate pattern analysis to investigate how brain patterns generalize over time. In our previous work (Bonetti et al. 2024), we demonstrated that distinguishing between previously memorized and systematically varied musical sequences resulted in consistent brain patterns persisting throughout the entire sequence duration. Specifically, our findings indicated that brain responses to individual sounds within memorized and novel sequences were consistently similar, suggesting ongoing monitoring of each sound, confirmation of predictions aligned with memory traces, and error detection when discrepancies occurred. In this study, we extended our investigation by comparing brain patterns between previously memorized melodies and entirely novel ones (i.e. not systematically varied after specific sounds) to determine whether differential brain activity patterns remained stable across the entire musical sequence or exhibited divergence. The findings of this study revealed no consistent patterns, indicating that the differential activity between corresponding tones of the memorized and novel sequences varied throughout the sequence (e.g. the differential activity at tone three differed from that at tones four or five). This discrepancy likely stems from two potential reasons. First, it may be attributed to the faster pace of each auditory stimulus (250 ms in the current study) compared to our previous investigation (350 ms in Bonetti et al. 2024). The increased tempo may necessitate a more holistic processing of the musical sequence by the brain, rather than focusing on individual sounds. Consequently, this could explain why different sounds are processed in distinct ways, thus preventing generalization over time in our decoding analysis. The alternative explanation involves the absence of systematic variations to the original sequences in the novel condition. In Bonetti et al. (2024), the novel melodies initially mirrored the previously memorized ones before gradually diverging. This divergence induced prediction errors, reflected in stable brain patterns over time, which suggested that the brain monitored the entire sequence in a similar manner. This might be because the brain recognized the beginning of the sequence and attempted to discern whether the change was temporary or marked the start of a varied melody. In the current study, where the novel melodies were entirely different from the memorized originals, the brain appeared not to engage in such continuous monitoring, as indicated by the nongeneralizable results of the multivariate pattern analysis.

Altogether, our findings can be seen in light of the global neuronal workspace hypothesis proposed by Dehaene and Changeux (2005). They defined the global workspace as a privileged network of brain areas, where conscious information is processed in terms of memory, attention, and valence and subsequently broadcast and made available to the whole-brain (Dehaene and Changeux 2005, 2011). As predicted by their hypothesis, the recognition of the memorized musical sequences extracted from Bach’s prelude—over and above the novel melodic sequences—led to stronger ignition of putative regions in the global workspace such as the hippocampus, cingulate gyrus, orbitofrontal cortex, and frontal operculum, perhaps reflecting the mechanisms that allow the brain to process, extract a meaningful representation, and recognize previously memorized musical sequences. Remarkably, our research did not only show the brain regions involved in the musical recognition task but also provided the dynamics of the activity of such regions, thus expanding the hypothesis proposed by Changeux. We observed that the brain activity linked to the recognition of memorized versus novel musical sequences significantly differed from the third tone of the sequences. Moreover, such different activity was first observed for the cingulate gyrus (third, fourth, and fifth tone of the melodies) and then for the hippocampal areas, inferior temporal cortex, insula, and frontal operculum (fourth and fifth tone of the melodies). Interestingly, our findings showed that the conscious, effortful recognition of temporal sequences involved several high-order brain areas, while previous studies on automatic recognition and prediction error associated with sudden deviations in auditory sequences (e.g. indexed by MMN and N100) revealed a major contribution of sensorial brain areas such as auditory cortices (Bonetti et al. 2021a; Bonetti et al. 2022b; Bonetti et al. 2018; Bonetti et al. 2017; Näätänen et al. 2007). This provides evidence for the relevance of the global neuronal workspace for conscious over automatic temporal sequence discrimination and recognition.

A further theory in the neuroscientific field that can be related to our results is predictive coding. In this framework, the brain is considered a generator of models of expectations of incoming stimuli. Recently, this theory has been linked to complex cognitive processes, finding a remarkable example in the neuroscience of music (Koelsch et al. 2019). In their work, Koelsch and colleagues suggested that the perception of music is the result of an active listening process where individuals constantly formulate hypothesis about the upcoming development of musical sentences, while those sentences are evolving and unfolding their ambiguities. Our study may be consistent with this perspective with regard to two of our outcomes. On the one hand, there is activity in the primary auditory cortex, responsible for the first sensorial processing of tones and decreasing over time. This may happen since the brain is predicting that a further tone will be presented, and its responses progressively decrease. On the other hand, the activation of brain areas related to memory and evaluative processes is increasing over time and stronger for the recognition of the memorized versus novel musical sequences. This may suggest that the brain has formulated predictions of the upcoming sounds based on the memory trace previously stored during the encoding part of our experimental task. The match between those predictions and the actual sounds presented to participants may lead to the activation of the brain areas that we observed in our experiment. However, further studies are required to provide additional evidence that can properly demonstrate whether the brain is predicting the upcoming tones of the musical melodies. In our research, the novel melodies were completely diverse from the memorized ones. Differently, future investigations may systematically vary the novel melodies, introducing the variations at specific times (e.g. from tone number two, from tone number three). If the brain is predicting the upcoming sound, it will systematically show a prediction error signal when the sequence is varied.

Analyzing the relationship between the brain activity during our recognition task and behavioral measures related to memory and musical skills showed very weak associations in small, isolated clusters. This suggested that having more engagement with music, general musical expertise, or a previous familiarity and higher appreciation for Bach’s prelude does not play a major role in modulating the brain activity during the musical recognition task. However, a mild yet interesting effect was observed for WM. This evidence, coherently with previous research (Bonetti et al. 2018), shows a connection between WM skills and neural data underlying memory tasks, indicating that the brain of individuals with higher WM abilities is characterized by a stronger activity when recognizing temporal sequences such as the excerpts from Bach’s prelude. Our results suggest that memory skills may be more important than musical abilities and expertise when recognizing temporal sequences, even when they consist of musical melodies. Future studies are called to further investigate the relationship between WM and the brain activity underlying recognition of long-term encoded auditory information.

We also investigated our data by focusing on MEG sensor analysis. In this regard, we found that brain activity was reflected by two ERF components: N100 to each sound and a slow negativity following the entire duration of the musical sequences. This negative waveform shares similarities with well-established ERF components such as contingent negative variation (CNV) (Walter et al. 1964; Naatanen et al. 2001). However, it also holds novel significance since in the current study it is associated with a conscious auditory recognition task, differently from classic studies on CNV (Walter et al. 1964; Rohrbaugh et al. 1976). Thus, in combination with findings reported by Bonetti et al. (2022a), our results offer new insights also into the dynamics of ERF and MEG sensor analyses. Future research is necessary to determine whether the observed slow negativity is influenced by the tempo of the stimuli or occurs independently of the musical pace.

Finally, it is important to highlight that while a major part of our findings was localized in the cerebral cortex (e.g. primary and secondary auditory cortex, insular cortex, cingulate cortex, frontal operculum), we also observed significant results reconstructed in deeper, subcortical areas such as hippocampus and basal ganglia. Whether MEG source reconstruction algorithms can reliably localize deep sources is part of a long-standing debate in the literature, and it is difficult to make definitive claims. On balance, it must be stated that deep sources are less easily detectable than cortical sources (Hillebrand and Barnes 2002; Goldenholz et al. 2009). Therefore, on the one hand, our results related to hippocampus and basal ganglia should be taken cautiously and call for future replications. On the other hand, there is no reason to believe that deep sources cannot be identified at all using MEG, as suggested by the mathematics behind source reconstruction algorithms such as beamforming and by several previous studies on MEG source reconstruction (Muller et al. 2019; Pizzo et al. 2019). To summarize, we argue that our subcortical results are reliable although obviously less accurate than our findings concerning cortical areas. Thus, they call for further confirmation by future studies, possibly employing not only MEG but also different machines and techniques such as fMRI and intracranial EEG (iEEG).

In conclusion, we have identified the spatiotemporal unfolding of fast-scale brain activity and functional connectivity associated with the recognition of previously memorized compared to novel musical sequences extended over time. We have shown the brain areas which were active and communicating during the processing of the subsequent items of the melodies, thus offering a first glimpse of the neural processing of temporal sequences. Future studies are called to replicate our results and further investigate the complex topic of encoding and recognition of temporal sequences. For instance, they should vary the experimental design and better disentangling the role of the different brain areas involved in the networks shown in our work.

## Supplementary Material

FigureSF1_bhae320

FigureSF2_bhae320

FigureSF3_bhae320

FigureSF5_bhae320

FigureSF6_bhae320

SupplementaryInformation_bhae320

## Data Availability

The code used for the full analysis pipeline is available at the following link: https://github.com/leonardob92/MelodiesRecognition_LB2017_BroadbandActivity_StaticFunctionalConnectivity.git Additional code related to the study is available at the following link: https://github.com/leonardob92/LBPD-1.0.git The multimodal neuroimaging data will be made available upon reasonable request.
