# Supplementary figures and images for: Spatiotemporal whole-brain activity and functional connectivity of melodies recognition
Source: Cereb Cortex. 2024 Aug 7;34(8):bhae320. doi: 10.1093/cercor/bhae320 (PMC11304985; doi:10.1093/cercor/bhae320)

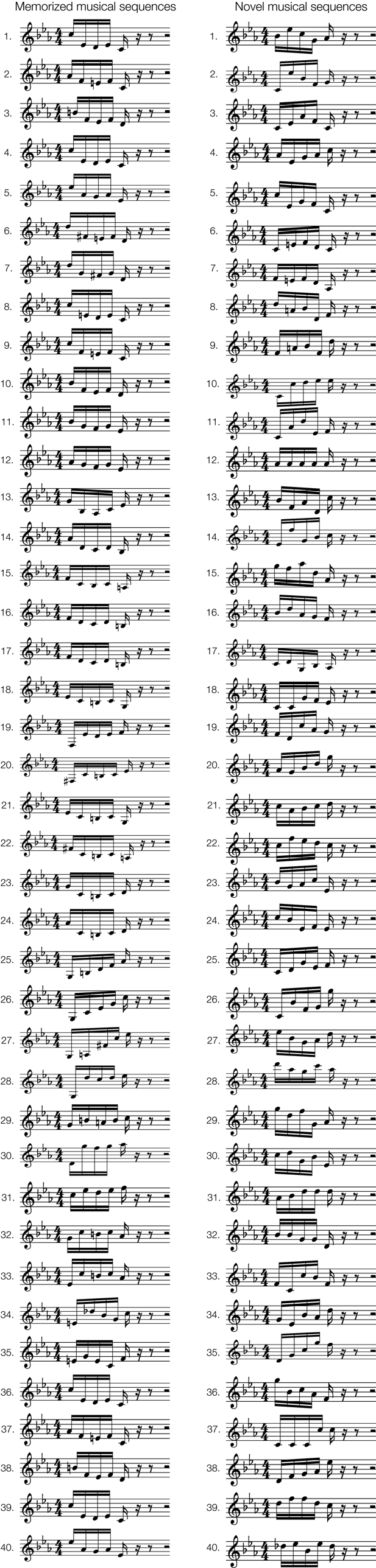

Supplement: FigureSF1_bhae320 [file figuresf1_bhae320.jpeg]

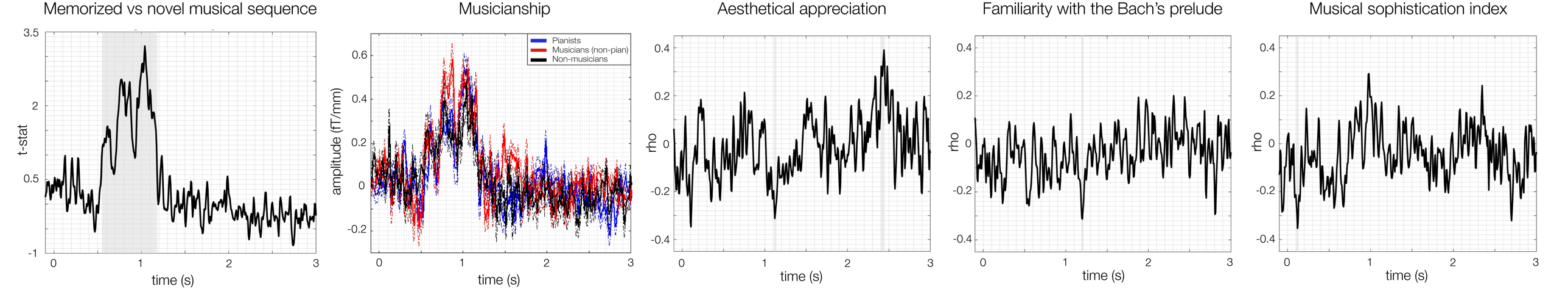

Supplement: FigureSF5_bhae320 [file figuresf5_bhae320.jpeg]

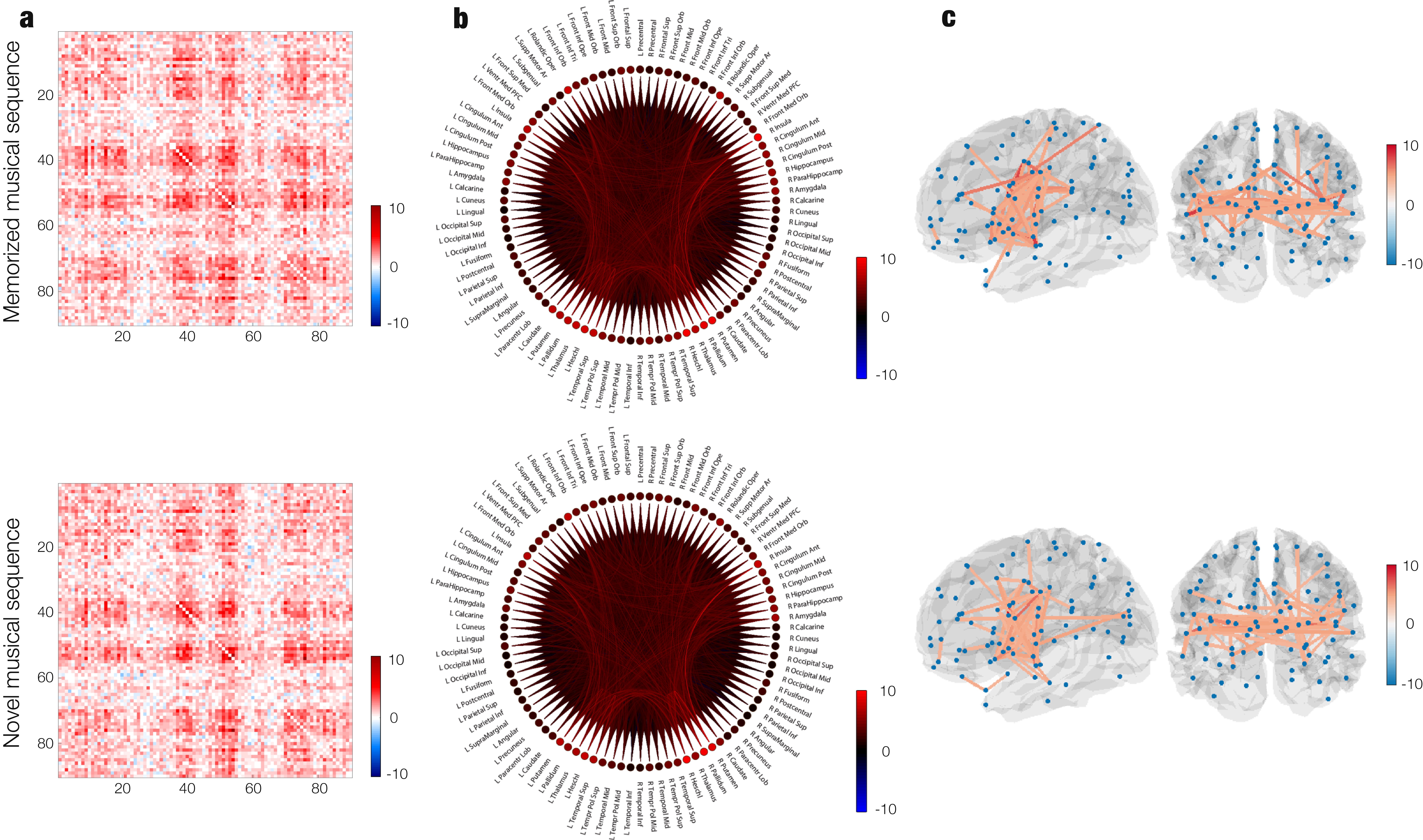

Supplement: FigureSF6_bhae320 [file figuresf6_bhae320.jpeg]
