# Supplementary material for: Spatiotemporal whole-brain activity and functional connectivity of melodies recognition
Source: Cereb Cortex. 2024 Aug 7;34(8):bhae320. doi: 10.1093/cercor/bhae320 (PMC11304985; doi:10.1093/cercor/bhae320)
Supplement: SupplementaryInformation_bhae320 [file supplementaryinformation_bhae320.pdf]

Supplementary information for

Spatiotemporal whole-brain activity and functional connectivity of melodies recognition

Bonetti L.[1,2,3*], Brattico, E. [1,6], Carlomagno, F. [1], Cabral J. [1,2,5], Stevner A. [1,2], Deco G.[4], Whybrow, P.C. [7], Pearce, M.[1], Pantazis, D.[8], Vuust P. [1] & Kringelbach M.L[1,2,3]

[1] *Center for Music in the Brain, Department of Clinical Medicine, Aarhus University & The Royal Academy of Music Aarhus/Aalborg, 8000, Denmark*

[2] *Centre for Eudaimonia and Human Flourishing, Linacre College, University of Oxford, United Kingdom*

[3] *Department of Psychiatry, University of Oxford, OX37JX, Oxford, United Kingdom*

[4] *Computational and Theoretical Neuroscience Group, Center for Brain and Cognition, Universitat Pompeu Fabra, Barcelona, Spain*

[5] *Life and Health Sciences Research Institute (ICVS), School of Medicine, University of Minho, 4710-057 Braga, Portugal*

[6] *Department of Education, Psychology, Communication, University of Bari Aldo Moro, 70121, Bari, Italy*

[7] *Semel Institute for Neuroscience and Human Behavior, University of California, CA 90095, Los Angeles, LA, USA*

[8] *McGovern Institute for Brain Research, Massachusetts Institute of Technology (MIT), MA 02139, Cambridge, USA*

**Corresponding author: Leonardo Bonetti, leonardo.bonetti@psych.ox.ac.uk*

SUPPLEMENTARY FIGURES

For high-resolution figures, please refer to the separate image files provided in the supplementary materials accompanying this article.

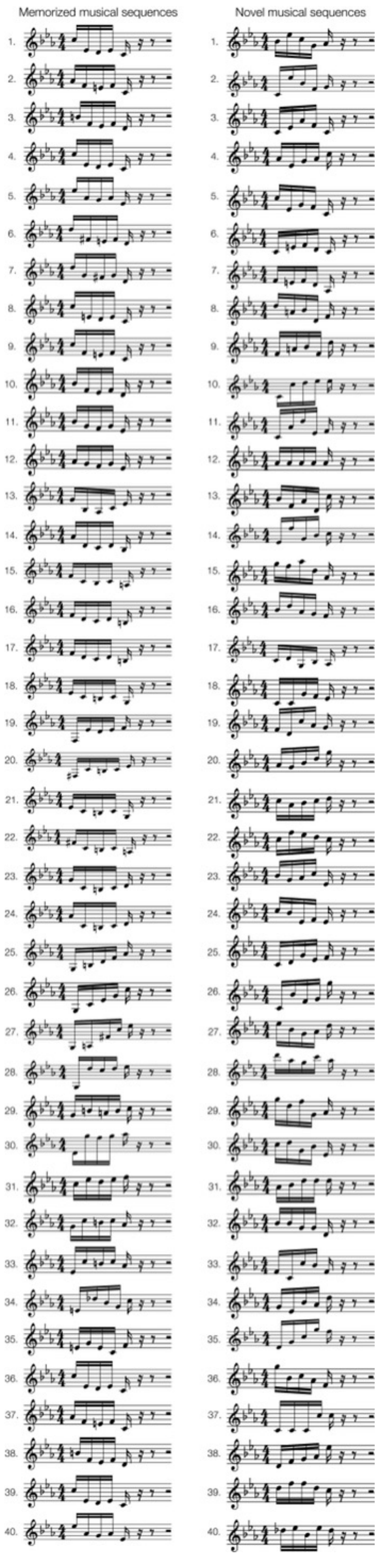
Memorized musical sequences
Novel musical sequences

Figure SF1. Musical sequences used in the experiment.

Depiction of all musical sequences used in the auditory 'old/new' paradigm employed in the study. On the left, we have reported the 40 musical sequences extracted from Bach's prelude (previously memorised musical sequences, 'old'). On the right, we have reported the 40 novel melodies (novel musical sequences, 'new') that were created and matched to Bach's prelude excerpts with regards to IC, H and main acoustic features.

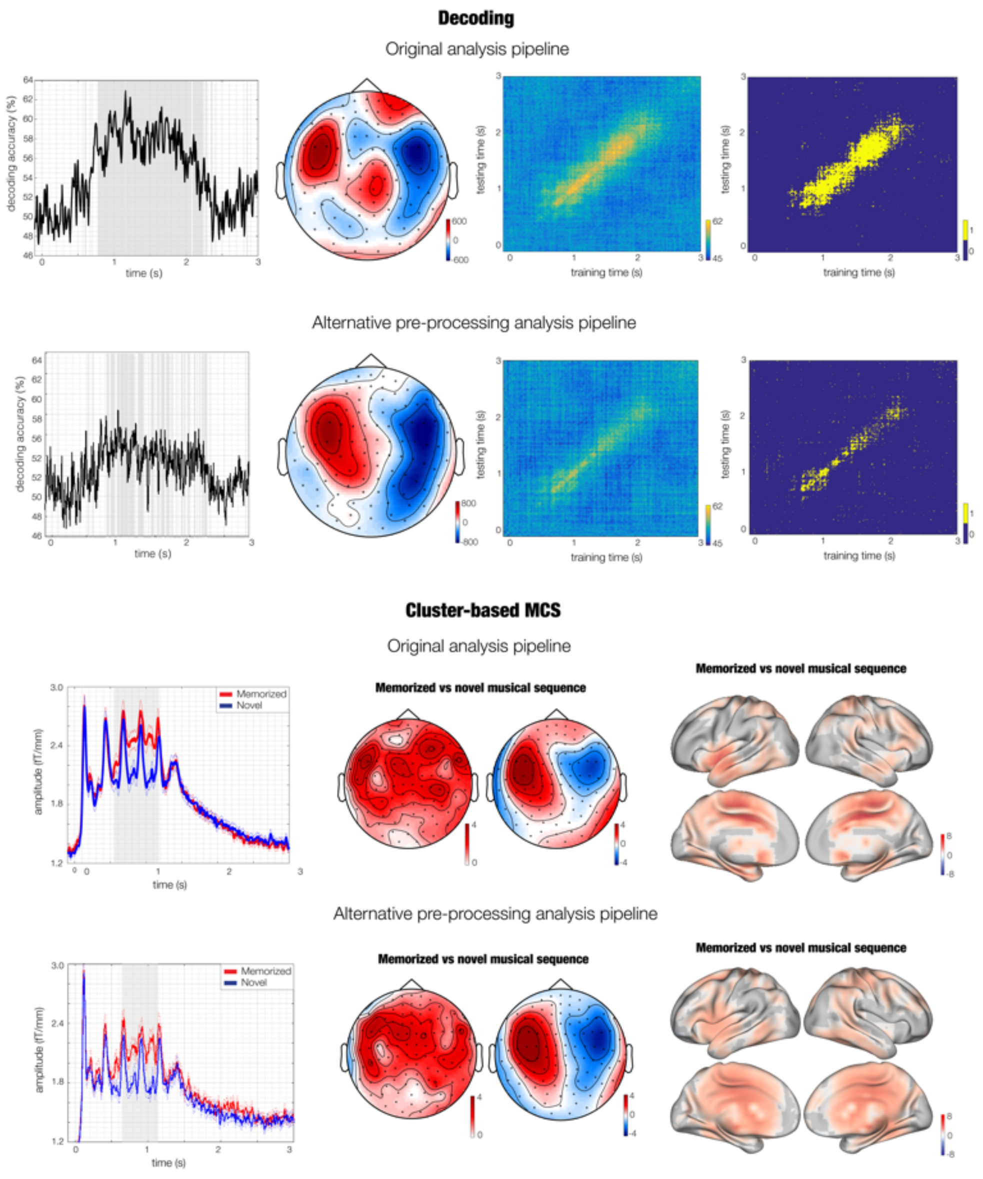

Figure SF2. Brain activity underlying memorised versus novel musical sequences: comparison between pre-processing pipelines. *The figure shows the results reported in the main text (labelled as 'Original analysis pipeline') versus the results obtained using a shortened pre-processing pipeline (labelled as 'Alternative pre-processing analysis pipeline) which consisted only of MaxFilter and ICA for removing eyeblink and heart-beat artefacts. The figure shows that the results are very similar.* ***Decoding –*** *Multivariate pattern analysis decoding the different neural activity associated with memorised versus novel musical sequences. Decoding time series (left, grey areas indicate significant time-points), spatial sequences depicted as topoplot (middle left), temporal generalisation decoding accuracy (middle right) and statistical output of significant prediction of training time on testing time (right).* ***Cluster-based MCS –*** *The left plot shows the amplitude associated with memorised (red) and novel musical sequences (blue). Grey areas show the time-points where the difference between memorised and novel sequences was significant. The middle plot illustrates a couple of topoplots showing brain activity for gradiometers (left, fT/cm) and magnetometers (right, fT) within the significant time-window emerged from the MCS. The values represent the statistics (t-values) contrasting the brain activity underlying recognition of*

memorised versus novel musical sequences. The right plot shows the neural sources for the recognition of the previously memorised versus novel musical sequences.

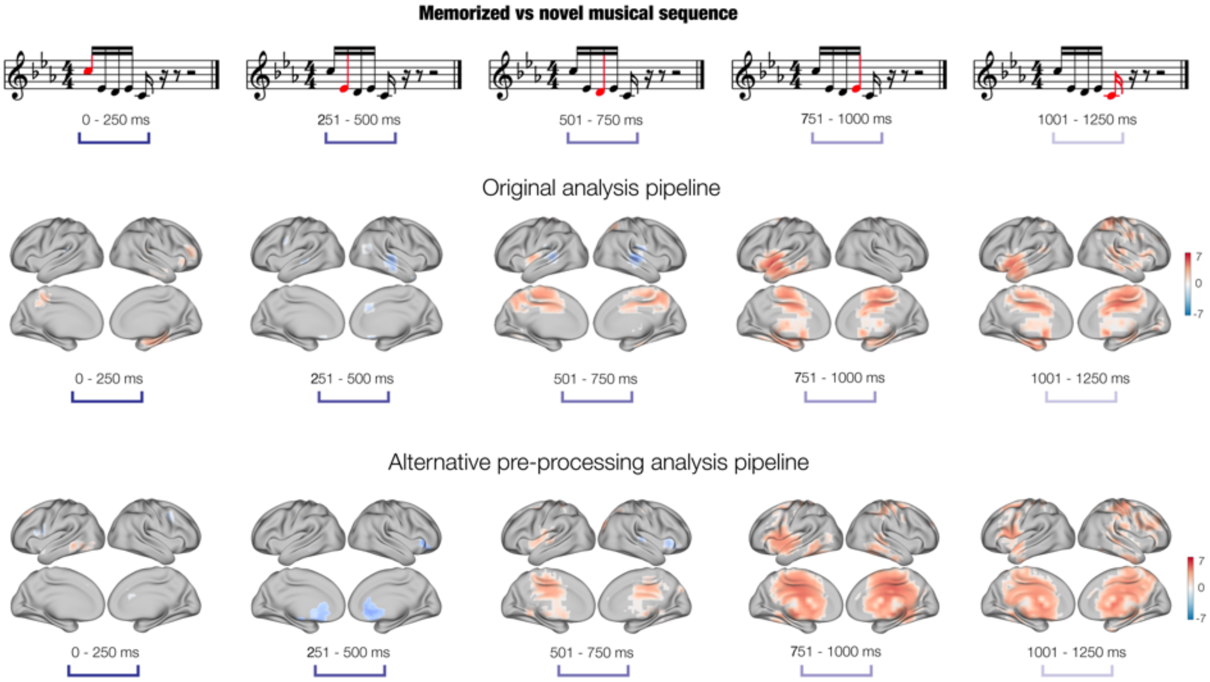

Figure SF3. Brain activity over time: comparison between pre-processing pipelines. *The figure shows the results reported in the main text (labelled as 'Original analysis pipeline') versus the results obtained using a shortened pre-processing pipeline (labelled as 'Alternative pre-processing analysis pipeline) which consisted only of MaxFilter and ICA for removing eyeblink and heart-beat artefacts. The figure shows that the results are very similar. The top row depicts an example trial for the memorised sequences. Red tones illustrate the dynamics of the musical excerpt. The second row (represented independently for the two pre-processing pipelines) indicates the brain activity (t-values) associated with the recognition of previously memorised versus novel musical sequences.*

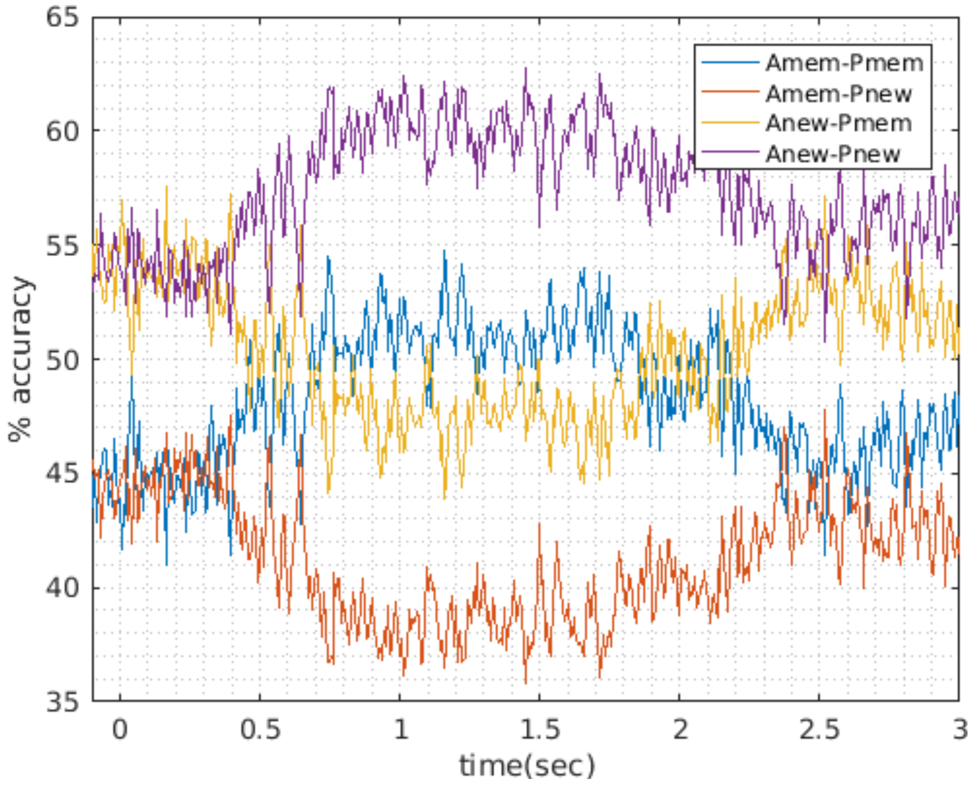

Figure SF4. Confusion matrix over time

Confusion matrix of the multivariate pattern analysis (decoding) computed for each time-point. The four time series show the decoding accuracy for the four following combinations: predicted memorised sequences - actual memorised sequences (i); predicted novel sequences - actual memorised sequences (ii); predicted memorised sequences - actual novel sequences (iii); predicted novel sequences - actual novel sequences (iv). The plot shows that the best decoding accuracy was for predicted novel sequences - actual novel sequences.

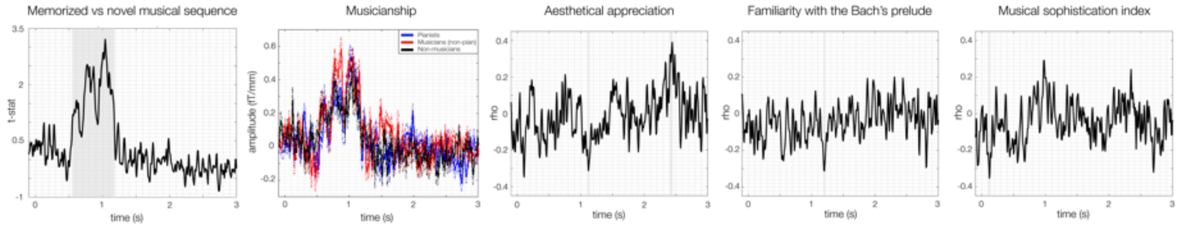

Figure SF5. Brain activity underlying auditory sequence recognition and musical skills.

The first plot from the left shows the contrast between brain activity underlying recognition of the previously memorised versus novel musical sequences. The second plot illustrates such contrast in relation to the musicianship groups of our sample (pianists, non-pianist musicians, non-musicians). The remaining plots show the correlation between the contrast and three measures of musical skills/features: aesthetical appreciation of the Bach's prelude (i), familiarity with the Bach's prelude (ii), general ability to be engaged with music and musical activities (iii).

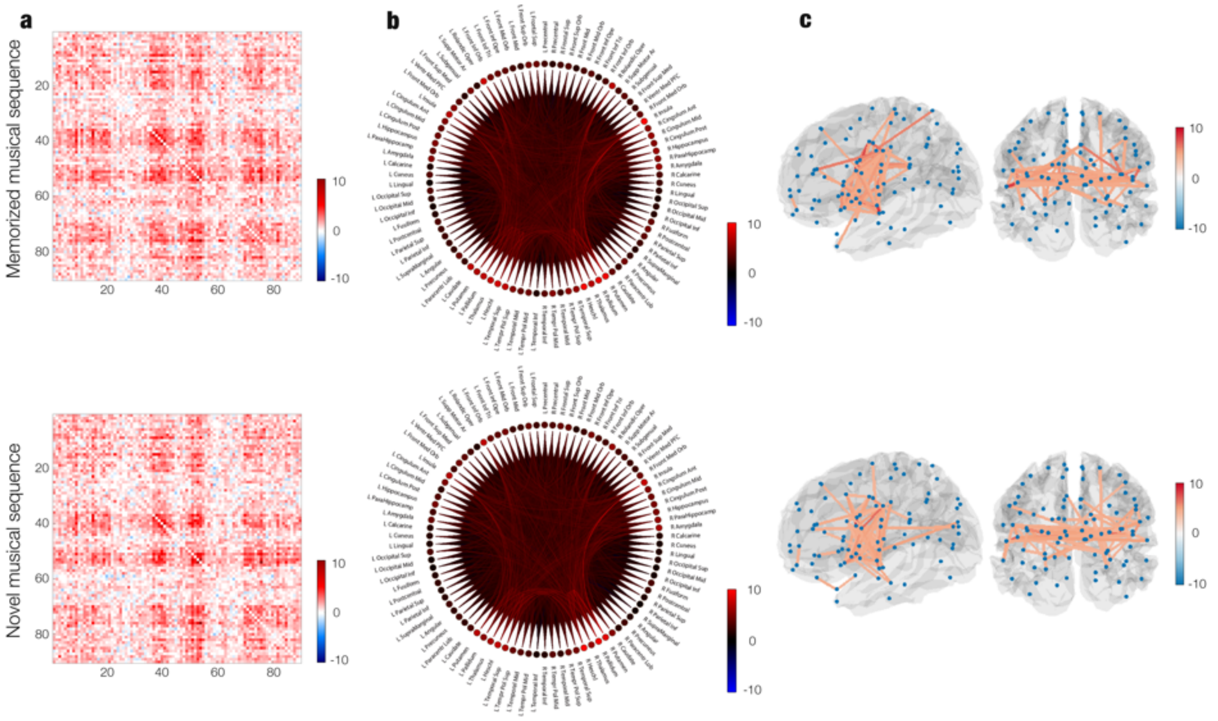

Figure SF6. Static functional connectivity for 2-8Hz. a – *Matrix representation of contrasts (t-values) between task versus baseline SFCs.* ***b*** *– Same values depicted in schemaballs.* ***c*** *– Same values depicted in brain templates. For each pair of brain templates, the left brain represents a left hemisphere perspective while the right one a posterior view of the brain.*

SUPPLEMENTARY TABLES

For supplementary tables too large to be included in this document, please refer to the corresponding Excel files available in the following GitHub repository:

https://github.com/leonardob92/MelodiesRecognition_LB2017_BroadbandActivity_StaticFunctionalConnectivity.git

Table ST1 – Memorised versus novel musical sequences – MEG sensors

**Cluster number**	**Size**	**Channels**	**Time-range (s)**	***p***
Gradiometers (memorised versus novel musical sequences)				
1	2117	90	0. 547 – 1.180	< .001
Magnetometers – positive (memorised versus novel musical sequences)				
1	817	24	0.627 – 1.180	< .001
Magnetometers – negative (memorised versus novel musical sequences)				
1	190	18	0.727 – 0.880	< .001
2	168	15	0.960 – 1.133	< .001

Table ST1*. Significant clusters of MEG sensors emerged from MCS contrasting memorised versus novel musical sequences. The table depicts these clusters independently for gradiometers and positive and negative magnetometers.*

Table ST2 – Novel versus memorised musical sequences – MEG sensors

**Cluster number**	**Size**	**Channels**	**Time-range (s)**	***p***
Gradiometers (novel versus memorised musical sequences)				
1	14	2	1.640 – 1.713	< .001
2	9	2	1.467 – 1.513	< .001
3	8	2	2.387 – 2.427	< .001
4	8	1	1.600 – 1.647	< .001
5	8	2	1.760 – 1.793	< .001
6	6	1	1.680 – 1.713	< .001
7	6	2	0.487 – 0.507	< .001
8	6	1	0.447 – 0.480	< .001

Table ST2. *Significant clusters of MEG sensors emerged from MCS contrasting novel versus memorised musical sequences. The table depicts these clusters for gradiometers.*

Table ST3 – Detailed information on significant clusters for MEG sensor data
Significant clusters of MEG sensors emerged from MCS contrasting memorised versus novel musical sequences. The table depicts those clusters with regards to significant channels and time-windows. The table also shows the results for the alternative pre-processing pipeline, comparing it with the original analysis pipeline.

Table ST4 – Detailed information on significant clusters for MEG source data
Significant clusters of MEG sources emerged from cluster-based permutation testing and related to memorised musical sequences versus baseline, novel musical sequences versus baseline and memorised versus novel musical sequences. The table depicts those clusters with regards to significant voxels, time-windows, and averaged t-values for each voxel. The table also shows the results for the alternative pre-processing pipeline, comparing it with the original analysis pipeline.

Table ST5 – Brain activity for each tone of the musical excerpts
Significant clusters of brain activity associated with each tone of the musical excerpts, reported for both conditions (memorised and novel musical sequences) and for their contrast (memorised versus novel musical sequences). The table also shows the results for the alternative pre-processing pipeline, comparing it with the original analysis pipeline.

SUPPLEMENTARY TEXT

SR1 – Additional information on Monte-Carlo simulations on MEG combined gradiometers and magnetometers

We employed a different approach by calculating several univariate t-tests and then correcting for multiple comparisons by using MCS. Before computing the t-tests, we averaged the trials over conditions, obtaining two mean trials, one for the memorised and one for the novel musical sequences. Then, we combined each pair of planar gradiometers by sum-root square. Afterwards, we computed a t-test for each combined planar gradiometer and each time-point in the time-range 0 – 2.500 seconds, contrasting the two experimental conditions. We reshaped the matrix for obtaining, for each time-point, a 2D approximation of the MEG channels layout that we binarized according to the *p*-values obtained from the previous t-tests (threshold = .01) and the sign of *t*-values. The resulting 3D matrix (*M*) was therefore composed by 0s when the t-test was not significant and 1s when it was. Then, to correct for multiple comparisons, we identified the clusters of 1s and assessed their significance by running MCS. Specifically, we made 1000 permutations of the elements of the original binary matrix *M*, identified the maximum cluster size of 1s and built the distribution of the 1000 maximum cluster sizes. Finally, we considered significant the original clusters that had a size bigger than the 99.9% maximum cluster sizes of the permuted data. Considering that magnetometers (differently from combined gradiometers) maintain the double polarity of the magnetic field, contrasting two experimental conditions presents potential technical ambiguities, admitting the theoretical possibility that two neighbouring clusters with opposite polarity and depicting different strengths between conditions (e.g. cond1 > cond2 (positive polarity) in cluster one and cond2 > cond1 (negative polarity) in cluster two) may be identified as one unique large (positive) cluster. For these reasons, at first, we carried out the algorithm by contrasting memorised versus novel musical sequences for combined planar gradiometers only. Then, on the basis of the significant clusters emerged, we used the same algorithm one more time for magnetometers only, within the significant time-range emerging from the first MCS (in this case: 0.547 – 1.180 seconds). This procedure allowed us to obtain more reliable and complete information about the different neural signal associated with the recognition of the memorised and novel musical sequences for both gradiometers and magnetometers. The whole MCS procedure was performed for memorised versus novel musical sequences and vice versa.

SR2 - Significantly central ROIs within the whole-brain network detected from the SFC matrices for delta, alpha, beta and gamma bands

We observed left cingulum middle ($p < 1.0e-07$), parahippocampal gyrus ($p = 5.5e-06$), amygdala ($p = 2.2e-06$), Heschl's gyrus ($p = 5.5e-06$), post-central gyrus ($p = 1.0e-04$), thalamus ($p < 1.0e-07$), right thalamus ($p = 2.2e-06$), pallidum ($p < 1.0e-07$), putamen ($p < 1.0e-07$), cuneus ($p < 1.0e-07$), caudate ($p = 3.3e-06$), cingulum anterior ($p < 1.0e-07$) and middle ($p = 7.7e-05$), insula ($p < 1.0e-07$) for delta; left precentral gyrus ($p = 8.8e-05$), cingulum middle ($p < 1.0e-07$) and posterior ($p = 1.1e-06$), hippocampus ($p < 1.0e-07$), Heschl's gyrus ($p < 1.0e-07$), right superior temporal gyrus ($p < 1.0e-07$), thalamus ($p = 6.6e-05$), Heschl's gyrus ($p < 1.0e-07$), caudate ($p = 5.0e-05$), cingulum middle ($p = 2.6e-05$), subgenual cortex ($p < 1.0e-07$), Rolandic operculum ($p < 1.0e-07$), fronto-superior orbital cortex ($p = 1.5e-05$), fronto-medial orbital cortex ($p = 1.8e-05$) for alpha; left supplementary motor area ($p = 5.6e-05$), cingulum middle ($p < 1.0e-07$), caudate ($p = 2.2e-06$), right temporal pole superior ($p = 1.1e-06$), Heschl's gyrus ($p = 1.1e-06$), thalamus ($p < 1.0e-07$), putamen ($p = 2.2e-06$), caudate ($p < 1.0e-07$), pallidum ($p = 4.4e-06$), parahippocampal gyrus ($p = 4.8e-06$), cingulum posterior ($p < 1.0e-07$), middle ($p < 1.0e-07$) and anterior ($p < 1.0e-07$), amygdala ($p = 1.1e-05$), fronto-medial orbital cortex ($p < 1.0e-07$), subgenual cortex ($p < 1.0e-07$) for beta; left cingulum anterior ($p < 1.0e-07$) and middle ($p < 1.0e-07$), caudate ($p < 1.0e-07$), putamen ($p < 1.0e-07$), pallidum ($p < 1.0e-07$), right pallidum ($p < 1.0e-07$), putamen ($p < 1.0e-07$), caudate ($p < 1.0e-07$), cingulum anterior ($p < 1.0e-07$), subgenual cortex ($p < 1.0e-07$), fronto-medial orbital cortex ($p < 1.0e-07$), precentral gyrus ($p < 2.2e-06$) for gamma.

These results are illustrated in **Figure 4**.
